# Global, regional, and national burden of lung cancer attributable to second-hand smoke from 1990 to 2021: a systematic analysis based on the Global Burden of Disease Study 2021

**DOI:** 10.3389/fonc.2025.1609230

**Published:** 2025-10-07

**Authors:** Zheng Zhang, Xiao-hui Zhang, Rong Huang, Jia-jia Fan, Xue-jiao Ma, Zi-han Jin, Qin Wang, Yu-long Li, Fei Su

**Affiliations:** ^1^ Graduate School, Beijing University of Chinese Medicine, Beijing, China; ^2^ Department of Integrative Oncology, China-Japan Friendship Hospital, Beijing, China; ^3^ Finance Department, China Press of Traditional Chinese Medicine Co.Ltd, Beijing, China

**Keywords:** second-hand smoke, lung cancer, epidemiology, disease burden, mortality, disability-adjusted life years

## Abstract

**Introduction:**

Second-hand smoke (SHS) exposure significantly contributes to lung cancer development, yet its global burden remains poorly quantified.

**Method:**

Using data from the Global Burden of Disease Study 2021, we analyzed SHS-related lung cancer deaths, disability-adjusted life-years (DALYs), and corresponding age-standardized rates (ASRs) across different regions and countries, stratified by gender, age, and Socio-Demographic Index (SDI). The estimated annual percentage changes (EAPCs) in the ASRs were calculated to determine the temporal trends spanning from 1990 to 2021. We also quantified the relationship between the SDI and the age-standardized rates of lung cancer caused by SHS.

**Results:**

In 2021, SHS accounted for approximately 0.10 million lung cancer deaths and 2.37 million DALYs worldwide, with a male-to-female mortality ratio of 1.38. The age-standardized mortality rate (ASMR) and age-standardized DALY rate (ASDR) manifested a trend of decline, with EAPC of -0.88 (95% UI: -0.94 to -0.82) and -1.25 (95% UI: -1.31 to -1. 19), respectively. The ASMR or ASDR peaked in middle and high-middle SDI regions, with a lower burden in other SDI regions. Nevertheless, the ASMR and ASDR in the high SDI region witnessed the most significant decline. Men bore a heavier burden of lung cancer attributable to SHS compared with women. The sexual disparity was more conspicuous among the elderly.

**Conclusion:**

During the past 32 years, the global burden of lung cancer attributable to SHS has revealed a downward tendency, concomitant with a decline in SHS exposure. The rise in absolute deaths and DALYs is driven by population growth and aging despite falling ASRs. Persistent epidemiological disparities across genders, age groups, and regions underscore the need for targeted interventions, particularly in middle and high-middle SDI settings.

## 1 Introduction

Lung cancer, as the most commonly diagnosed malignant tumor, stands as the primary cause of cancer-related deaths on a global scale. Drawing on the latest data from GLOBOCAN 2022, roughly 2.5 million individuals were newly diagnosed with lung cancer, constituting 12.4% of the global cancer cases. In the same year, approximately 1.8 million people worldwide succumbed to lung cancer, representing 18.7% of the total global cancer deaths (1). In addition, lung cancer holds the 17th position among the leading causes of disability-adjusted life-years (2). In terms of economic impact, it is forecasted that the total financial cost globally associated with lung cancer from 2020 to 2050 will reach 3.9 trillion dollars, equivalent to 0.085% of the total GDP (3). Consequently, alleviating the substantial health, economic, and social burdens imposed by lung cancer has become an urgent issue that requires immediate resolution within the domain of public health.

Exposure to second-hand smoke (SHS) from tobacco products is a crucial risk factor in the pathogenesis of various diseases. SHS comprises “mainstream smoke” exhaled by smokers and “sidestream smoke” generated from burning tips of cigarettes, cigars, or pipes (4). Although the implementation of smoke-free laws has contributed to a reduction in the global smoking prevalence, 79% of the worldwide population remains unshielded by smoke-free legislation (5). It is estimated that SHS exposure led to 1.3 million deaths in 2021 globally, inflicting colossal economic losses (6). Tobacco smoke contains more than 60 carcinogens, which the International Agency for Research on Cancer (IARC) has classified into group 1 and group 2A carcinogens (7). Despite active smoking continuing to be the dominant etiology for lung cancer, research has revealed that exposure to SHS can induce substantial sub-clonal mutations in genes with APOBEC-type signatures to facilitate the process of lung carcinogenesis (8). A multitude of epidemiological evidence has indicated that exposure to SHS augments the risk of lung cancer onset by over 20% (9–11). The global burden of lung cancer attributable to secondhand smoke (SHS) exhibits significant regional variation, with inconsistencies in SHS definitions across epidemiological studies (12, 13). Therefore, it remains a daunting challenge to comprehensively elucidate the impacts of SHS exposure duration and intensity on a global scale.

Given the significant disease burden imposed by lung cancer and the carcinogenic hazards of SHS, it is of great importance to comprehensively elucidate the disease burden of lung cancer caused by SHS, which will guide tobacco control management and provide guidance for the prevention of lung cancer. To date, the epidemiological profiles of the disease burden of lung cancer attributable to SHS have not been comprehensively estimated across global, regional, and national scales. There is only one article that reported the disease burden attributable to SHS based on the Global Burden of Disease, Injuries, and Risk Factors Study (GBD) 2021. Notably, it regrettably lacks a systematic evaluation of the burden of SHS-related lung cancer and its spatiotemporal trends (12). Moreover, the current literature lacks in-depth research on the association between SHS-related lung cancer and the socio-demographic index (SDI). The GBD 2021 represents a recent and comprehensive global epidemiological study aimed at measuring the burden caused by diseases, injuries, and risk factors across countries and territories worldwide. This study employed the latest GBD 2021 dataset and the explicit SDI-based evaluation to quantify the disease burden of SHS-related lung cancer and its spatiotemporal trends from 1990 to 2021. We analyzed the data by region, gender, and age group and explored the link between the disease burden and SDI. These findings could offer valuable insights to develop public health policies to control SHS and reduce the lung cancer burden.

## 2 Methods

### 2.1 Study data

The data for this study originated from the GBD 2021 study, which comprehensively offers updated epidemiological estimates for 371 diseases and injuries and 88 risk factors across 21 GBD regions of 204 countries and territories from 1990 to 2021. We extracted lung cancer burden estimates attributable to SHS from the Global Health Data Exchange (GHDx) platform using the GBD Results Tool (https://vizhub.healthdata.org/gbd-results/). Our analysis specifically examined risk-outcome pairs linking SHS exposure to lung cancer. We obtained mortality, disability-adjusted life-years (DALYs), age-standardized mortality rate (ASMR), and age-standardized DALY rate (ASDR) for SHS-related lung cancer across 204 countries and territories during the study period.

### 2.2 Definition

The Global Burden of Disease (GBD) Study classifies lung cancer as tumors originating within the trachea, bronchi, or lungs, designated by codes C33 and C34-C34.92 in the 10th edition of the International Classification of Diseases (ICD-10). The equivalent ICD-9 codes include 162-162.9, 209.21, V10. 1-V10.20, V16. 1-V16.2, and V16.4-V16.40 (2). Exposure to SHS refers to involuntary inhalation of tobacco smoke in domestic or occupational settings. The GBD 2021 data specifically captures SHS exposure among non-smokers, encompassing former smokers and occasional smokers, using self-reported measures (12, 14). The socio-demographic index (SDI) in GBD 2021 quantifies regional social and demographic development through three components: the total fertility rates among women under 25, mean educational attainment in those aged 15+, and lag-adjusted per capita income. This index ranges from 0 to 1, with higher values indicating greater socioeconomic development. The 204 countries and regions are stratified into five distinct categories: high SDI (>0.81), high-middle SDI (0.70–0.81), middle SDI (0.61–0.69), middle-low SDI (0.46–0.60), and low SDI (<0.46). Moreover, these countries and regions are grouped into 22 GBD regions by their socioeconomic resemblances and geographical adjacencies.

### 2.3 Estimation of SHS-related lung cancer burden

Methods for assessing lung cancer have been extensively discussed in prior academic literature (2, 15). In this study, we provided a comprehensive overview of the computational methods employed in the GBD 2021. Data on lung cancer mortality were obtained from various sources, including cancer registries, civil vital registration systems, and verbal autopsies. In places where mortality data were unavailable, the mortality-to-incidence ratio model was used to derive mortality estimates from incidence data. Taking into account factors such as location, year, and age, these data were incorporated into a Cause of Death Ensemble Model (CODEm) to estimate mortality. Disability-adjusted life-years (DALYs) serve as the optimal metric for quantifying disease burden, which are composed of years of life lost (YLLs) due to premature death and years lived with disability (YLDs). YLLs are derived from multiplying lung cancer deaths within an age cohort by the remaining life expectancy of the population. YLDs result from the product of lung cancer prevalence and severity-specific disability weights. Under the framework of comparative risk analysis, exposure data were modeled using spatiotemporal Gaussian process regression or DisMod-MR 2.1 (Disease Modelling Meta-Regression; version 2. 1). An age–gender–location–year–risk factor exposure model was constructed to generate quantitative relative risk estimates for each risk–outcome pair. Subsequently, these estimates were matched with the corresponding exposure estimates to calculate the population attributable fraction (PAF) for each risk–outcome pair. Finally, the PAF was multiplied by the corresponding outcome rate to obtain the estimates of lung cancer mortality or DALYs attributable to SHS exposure. The specific calculation procedures can be found in previous studies (14).

### 2.4 Statistical analysis

Age-standardized rates (ASRs) enable comparisons of mortality and DALY rates between countries with differing age structures and population characteristics. The estimated annual percentage change (EAPC) quantifies the trends in ASRs from 1990 to 2021. The EAPC measures the average annual percentage change in a specific indicator over time. This metric represents the slope of the linear relationship between the Napierian logarithm of the indicator and time, expressed as y = α + βx + ϵ, where x corresponds to the year and y equals ln(ASR). The logarithmic transformation of the ASRs facilitates modeling relative rate changes over time while maintaining a linear trend pattern. The EAPC values and corresponding 95% confidence intervals (CIs) were calculated using the formula 100 × [Exp(β) - 1]. We verified the log-linear assumption for EAPC. When both the EAPC and its 95% confidence interval (CI) exceed 0, the ASRs exhibit an increasing trend, whereas values below 0 indicate a decreasing trend. Gaussian process regression and locally estimated scatterplot smoothing smoother models assessed the relationship between SDI, ASR, and EAPC. Spearman’s rank correlation test evaluated these associations. All rates were standardized per 100,000 residents, with 95% uncertainty intervals reported for all estimates. Statistical significance was set at a two-sided *P*-value <0.05. Data processing, analyses, and visualizations were performed using R version 4.4.2.

## 3 Results

### 3.1 Global variations and trends in lung cancer burden attributable to second-hand smoke

**Figure 1:**
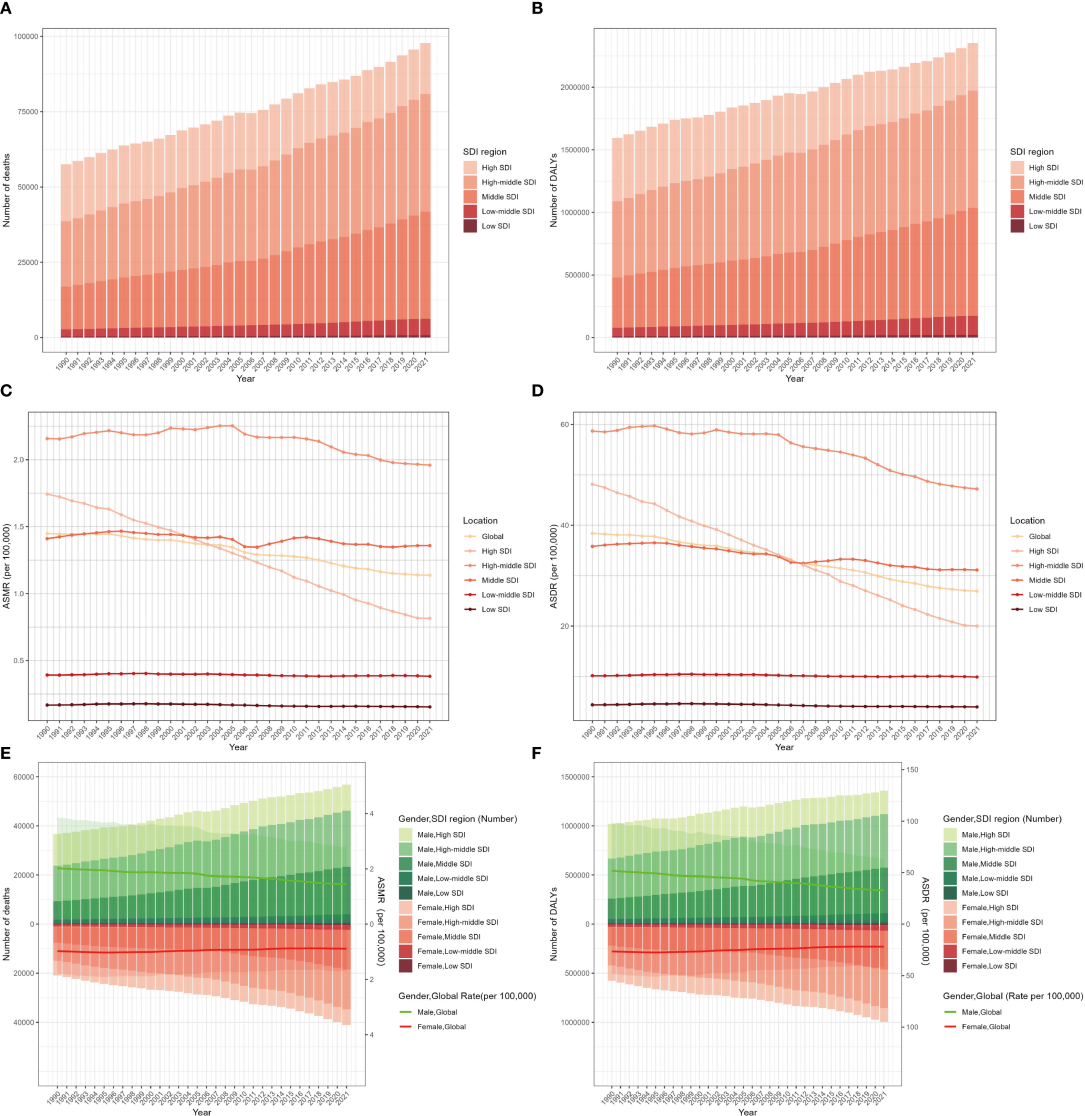
Global burden of lung cancer deaths **(A)**, DALYs **(B)**, ASMR **(C)**, and ASDR **(D)** attributable to second-hand smoke for both sexes from 1990 to 2021 by SDI region. Deaths and ASMR **(E)** and DALYs and ASDR **(F)** of lung cancer attributable to second-hand smoke from 1990 to 2021 by sex and SDI region. The bars represent the deaths and DALY numbers of lung cancer attributable to second-hand smoke. The lines with 95% UI display ASMR and ASDR attributable to second-hand smoke. ASMR, age-standardized mortality rate; ASDR, age-standardized DALY rate; DALYs, disability-adjusted life-years; UI, uncertainty interval; SDI, sociodemographic index.

On a global scale, exposure to SHS was associated with approximately 97,911 lung-cancer-related deaths, with DALYs totaling approximately 2.36 million correspondingly in 2021. The male–female ratio of lung cancer deaths and DALYs attributable to second-hand smoke was almost 1.38 in 2021. Between 1990 to 2021, it was revealed that there was an apparent increase in the number of lung cancer deaths and DALYs due to SHS. Nevertheless, the ASMR and ASDR manifested slight decreases, with EAPC of -0.88 (95% CI: -0.94 to -0.82) and -1.25 (95% CI: -1.31 to -1.19), respectively (**Table 1**, **Figures 1C, D**). These decreasing trends were significantly more pronounced in men than women (**Table 1**, **Figures 1E, F**).

**Table 1:** Global and regional lung cancer burden attributable to second-hand smoke in 1990 and 2021 and EAPCs of both ASMR and ASDR from 1990 to 2021.

Characteristics	1990				2021				EAPC	
	Death cases (95% UI)	ASMR (95% UI)	DALYs (95% UI)	ASDR (95% UI)	Death cases (95% UI)	ASMR (95% UI)	DALYs (95% UI)	ASDR (95% UI)	ASMR (95% UI)	ASDR (95% UI)
Global	57,618 (7,083–107,842)	1.45 (0.18–2.72)	1,598,871 (196,922–2,982,788)	38.4 (4.72–71.68)	97,911 (11,955–184,913)	1.14 (0.14–2.15)	2,355,866 (290,211–4,442,996)	26.93 (3.32–50.83)	-0.88 (-0.94 to -0.82)	-1.25 (-1.31 to -1.19)
Sex										
Male	36,764 (4,318–69,861)	2.03 (0.24–3.86)	1,020,918 (120,602–1,938,069)	51.88 (6.11–98.51)	56,848 (6,655–109,071)	1.44 (0.17–2.78)	1,359,557 (158,640–2,596,453)	32.88 (3.85–62.82)	-1.07 (-1.15 to -1)	-1.48 (-1.55 to -1.4)
Female	20,854 (2,762–37,840)	0.98 (0.13–1.78)	577,953 (76,274–1,045,073)	26.56 (3.5–48.03)	41,063 (5,536–78,059)	0.89 (0.12–1.69)	996,309 (135,988–1,907,079)	21.81 (2.98–41.75)	-0.57 (-0.65 to -0.49)	-0.89 (-0.97 to -0.81)
SDI region										
High SDI	18,917 (2,323–36,155)	1.74 (0.21–3.33)	507,723 (62,348–966,857)	48.12 (5.91–91.54)	16,945 (2,216–32,743)	0.82 (0.11–1.58)	380,285 (49,959–738,240)	19.99 (2.63–38.76)	-2.57 (-2.67 to -2.46)	-2.93 (-3.04 to -2.81)
High-middle SDI	21,702 (2,611–40,729)	2.16 (0.26–4.04)	609,304 (73,612–1,146,504)	58.69 (7.08–110.37)	39,124 (4,613–73,341)	1.96 (0.23–3.67)	936,577 (111,577–1,736,627)	47.2 (5.65–87.54)	-0.36 (-0.46 to -0.25)	-0.78 (-0.89 to -0.67)
Middle SDI	14,192 (1,799–25,709)	1.41 (0.18–2.56)	401,195 (50,957–727,467)	35.8 (4.54–64.87)	35,511 (4,497–67,457)	1.36 (0.17–2.58)	862,344 (110,218–1,626,035)	31.13 (3.97–58.93)	-0.24 (-0.3 to -0.18)	-0.58 (-0.63 to -0.52)
Low-middle SDI	2,350 (311–4,440)	0.39 (0.05–0.74)	67,474 (8,771–127,412)	10.15 (1.33–19.21)	5,478 (699–10,459)	0.38 (0.05–0.73)	152,292 (19,444–291,023)	9.9 (1.26–18.89)	-0.13 (-0.17 to -0.09)	-0.13 (-0.17 to -0.09)
Low SDI	374 (43–740)	0.17 (0.02–0.33)	10,837 (1,223–21,437)	4.37 (0.5–8.65)	770 (94–1,556)	0.15 (0.02–0.31)	22,327 (2,712–45,068)	3.96 (0.48–8.01)	-0.43 (-0.52 to -0.35)	-0.49 (-0.58 to -0.4)
GBD region										
High-income Asia Pacific	2,639 (344–5,051)	1.32 (0.17–2.53)	65,961 (8,573–125,048)	32 (4.16–60.71)	3,440 (436–6,957)	0.68 (0.09–1.4)	62,673 (7,816–128,139)	14.83 (1.86–30.46)	-2.41 (-2.64 to -2.19)	-2.74 (-2.97 to -2.51)
High-income North America	7,019 (857–13,570)	2.09 (0.25–4.03)	188,134 (22,906–360,573)	58.68 (7.12–111.97)	4,568 (588–9,059)	0.7 (0.09–1.38)	107,413 (13,713–211,496)	17.36 (2.21–34.11)	-3.75 (-3.9 to -3.6)	-4.11 (-4.26 to -3.96)
Western Europe	9,027 (1,054–17,210)	1.65 (0.19–3.14)	244,694 (28,689–466,150)	46.96 (5.53–89.32)	6,227 (775–12,307)	0.73 (0.09–1.43)	150,799 (19,175–295,049)	19.46 (2.49–38.01)	-2.59 (-2.68 to -2.49)	-2.79 (-2.91 to -2.67)
Australasia	284 (30–604)	1.22 (0.13–2.61)	7,576 (805–16,022)	33.37 (3.56–70.55)	242 (26–526)	0.47 (0.05–1.01)	5,814 (611–12,570)	12.12 (1.27–26.13)	-3.04 (-3.14 to -2.95)	-3.2 (-3.28 to -3.12)
Andean Latin America	48 (6–91)	0.24 (0.03–0.45)	1,323 (168–2,503)	6.06 (0.77–11.48)	67 (8–132)	0.11 (0.01–0.22)	1,726 (201–3,400)	2.84 (0.33–5.59)	-2.9 (-3.18 to -2.63)	-3.02 (-3.31 to -2.72)
Tropical Latin America	916 (112–1,758)	1.02 (0.12–1.96)	25,378 (3,109–48,617)	26.02 (3.18–49.85)	1,368 (161–2,745)	0.53 (0.06–1.07)	34,368 (4,093–69,074)	13.08 (1.56–26.27)	-2.3 (-2.4 to -2.19)	-2.44 (-2.53 to -2.34)
Central Latin America	338 (42–634)	0.43 (0.05–0.8)	8,867 (1,096–16,588)	10.18 (1.26–19.06)	455 (55–866)	0.18 (0.02–0.35)	11,230 (1,346–21,277)	4.39 (0.53–8.31)	-3 (-3.12 to -2.89)	-3.02 (-3.14 to -2.9)
Southern Latin America	692 (87–1,406)	1.49 (0.19–3.03)	19,432 (2,494–39,639)	41.45 (5.33–84.54)	614 (74–1,314)	0.71 (0.08–1.52)	15,436 (1,845–33,085)	18.3 (2.18–39.2)	-2.31 (-2.53 to -2.09)	-2.57 (-2.81 to -2.33)
Caribbean	269 (34–535)	1.07 (0.13–2.13)	6,280 (799–12,386)	23.99 (3.05–47.31)	357 (38–727)	0.66 (0.07–1.34)	8,086 (863–16,491)	14.96 (1.6–30.52)	-1.56 (-1.73 to -1.39)	-1.59 (-1.78 to -1.4)
Central Europe	3,808 (480–7,293)	2.5 (0.32–4.79)	110,561 (13,890–211,809)	72.72 (9.13–139.01)	3,494 (438–6,729)	1.61 (0.2–3.1)	86,225 (10,669–165,607)	42.31 (5.22–81.04)	-1.46 (-1.64 to -1.27)	-1.8 (-2.01 to -1.59)
Eastern Europe	4,323 (561–8,351)	1.5 (0.19–2.9)	125,653 (16,106–243,121)	43.66 (5.59–84.28)	2,827 (354–5,554)	0.8 (0.1–1.58)	75,436 (9,497–147,003)	22.23 (2.83–43.42)	-2.1 (-2.26 to -1.93)	-2.3 (-2.47 to -2.13)
Central Asia	709 (92–1,415)	1.45 (0.19–2.9)	21,351 (2,754–42,678)	41.95 (5.4–83.79)	600 (78–1,192)	0.73 (0.09–1.44)	16,793 (2,175–33,398)	18.75 (2.44–37.2)	-1.8 (-1.95 to -1.65)	-2.24 (-2.36 to -2.12)
North Africa and Middle East	2,151 (237–4,062)	1.31 (0.15–2.46)	60,176 (6,579–114,545)	33.1 (3.63–62.82)	4,534 (526–8,819)	1.04 (0.12–2.03)	120,069 (13,861–232,534)	24.83 (2.88–48.46)	-0.78 (-0.89 to -0.66)	-0.99 (-1.09 to -0.89)
South Asia	1,481 (186–2,775)	0.26 (0.03–0.5)	42,798 (5,403–79,876)	6.78 (0.85–12.68)	3,869 (526–7,497)	0.26 (0.04–0.51)	108,253 (14,692–210,038)	6.85 (0.93–13.28)	-0.42 (-0.6 to -0.24)	-0.35 (-0.52 to -0.17)
Southeast Asia	2,122 (291–3,992)	0.86 (0.12–1.63)	59,389 (8,258–112,014)	21.65 (2.99–40.78)	5,326 (696–10,098)	0.83 (0.11–1.57)	142,237 (18,519–271,986)	20.32 (2.66–38.74)	-0.33 (-0.42 to -0.24)	-0.41 (-0.5 to -0.32)
East Asia	21,359 (2,640–40,336)	2.6 (0.32–4.9)	598,410 (74,708–1,128,102)	64.41 (7.99–121.28)	59,196 (7,267–111,539)	2.75 (0.34–5.18)	1,387,975 (172,611–2,594,496)	62.42 (7.79–116.4)	0.14 (0.01 to 0.26)	-0.19 (-0.29 to -0.08)
Oceania	18 (2–37)	0.67 (0.08–1.41)	492 (57–1,030)	15.9 (1.83–33.37)	51 (7–113)	0.75 (0.1–1.66)	1,411 (179–3,141)	17.75 (2.26–39.22)	0.3 (0.23 to 0.38)	0.29 (0.21 to 0.37)
Western Sub-Saharan Africa	64 (8–125)	0.07 (0.01–0.14)	1,781 (227–3,460)	1.89 (0.24–3.68)	142 (18–278)	0.07 (0.01–0.15)	3,995 (492–7,784)	1.85 (0.23–3.61)	-0.05 (-0.13 to 0.03)	-0.15 (-0.23 to -0.06)
Eastern Sub-Saharan Africa	91 (11–179)	0.12 (0.01–0.23)	2,737 (336–5,391)	3.24 (0.4–6.4)	142 (18–272)	0.08 (0.01–0.16)	4,306 (533–8,218)	2.21 (0.27–4.22)	-1.36 (-1.45 to -1.26)	-1.48 (-1.59 to -1.38)
Central Sub-Saharan Africa	38 (5–79)	0.16 (0.02–0.34)	1,168 (143–2,457)	4.47 (0.54–9.33)	84 (10–180)	0.14 (0.02–0.31)	2,671 (297–5,691)	3.99 (0.45–8.51)	-0.44 (-0.75 to -0.13)	-0.42 (-0.73 to -0.11)
Southern Sub-Saharan Africa	223 (27–440)	0.81 (0.1–1.6)	6,711 (821–13,291)	22.55 (2.75–44.64)	307 (40–584)	0.52 (0.07–0.99)	8,950 (1,160–17,074)	14.07 (1.82–26.85)	-1.48 (-1.7 to -1.27)	-1.56 (-1.78 to -1.35)

ASMR, age-standardized mortality rate; ASDR, age-standardized DALY rate; DALYs, disability-adjusted life-years; UI, uncertainty interval; EAPC, estimated annual percentage change; CI, confidence interval; GBD, global burden of disease; SDI, sociodemographic index.

### 3.2 Spatiotemporal burden of lung cancer attributable to second-hand smoke by regions

The global burden of lung cancer attributable to SHS in 2021 varied substantially across SDI regions. The high-middle SDI region accounted for the largest share of SHS-related lung cancer deaths (0.39 million) and DALYs (0.94 million), representing over one-third of the global total in 2021 (**Table 1**, **Figures 1A, B**). Between 1990 and 2021, it was only the high SDI region that experienced a downward trend in SHS-attributable lung cancer deaths and DALYs, with the number of DALYs decreasing from 0.51 million to 0.38 million (**Table 1**, **Figures 1C, D**). Meanwhile, the high-middle region possessed the highest ASMR and ASDR, while the low SDI region had the lowest ASMR and ASDR. The ASMR and ASDR revealed a declining trend in all SDI regions from 1990 to 2021. The ASMR (EAPC: -2.57, 95% CI: -2.67 to - 2.46) and ASDR (EAPC: -2.93, 95% CI: -3.04 to -2.81) in the high SDI region witnessed the most significant decline (**Table 1**, **Supplementary Figures S1A, B**). While gender disparities in ASMR and ASDR persisted across all SDI regions, these gaps narrowed over time except in middle SDI regions. Notably, high-middle SDI region was the only region where the ASMR and ASDR among women increased (**Table 1**, **Supplementary Figures S2A, B**). It might be due to the slower implementation of tobacco control measures in these areas as well as the fact that women are more likely to be exposed to tobacco smoke in the domestic environment.

**Figure 2:**
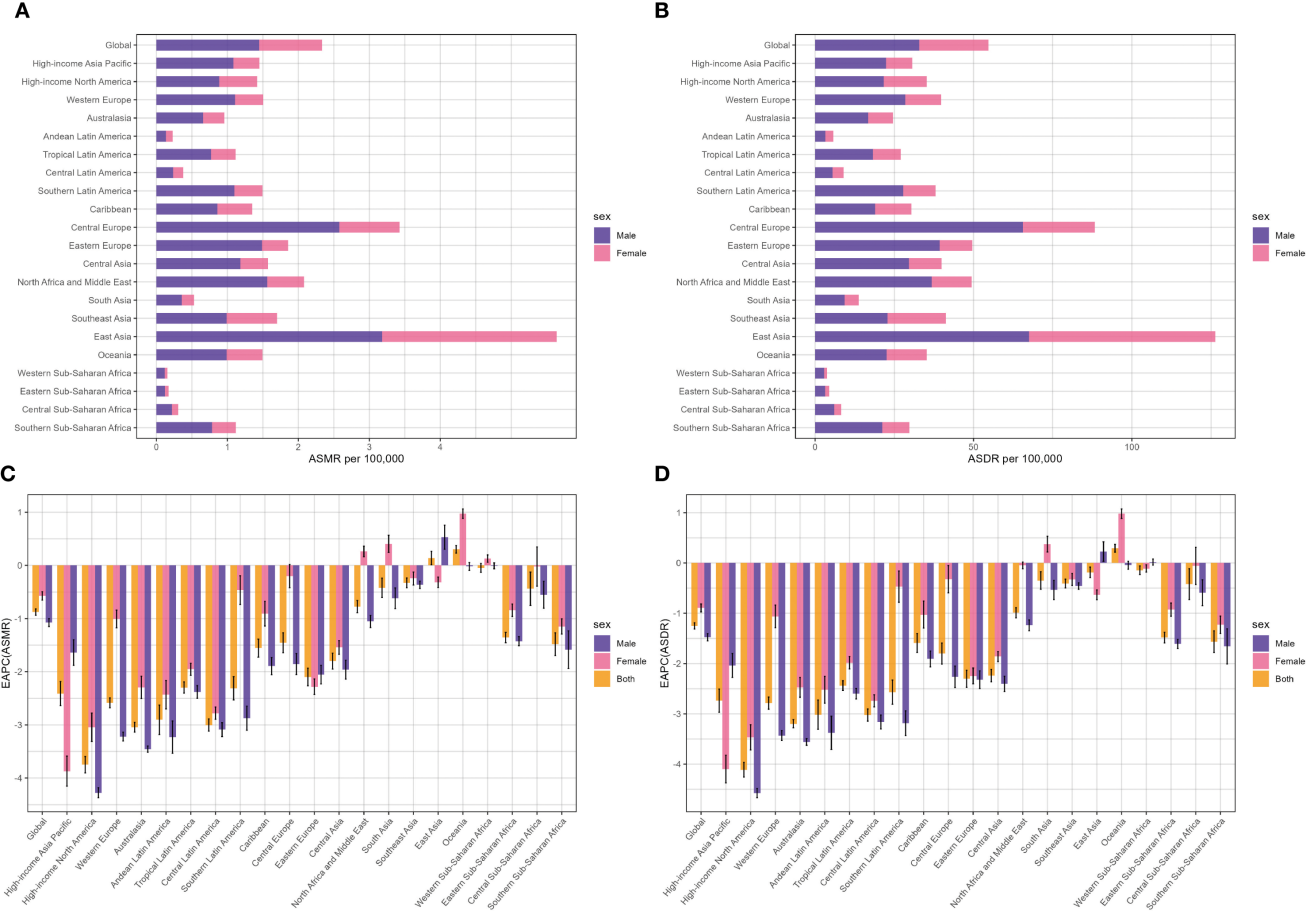
Distribution of lung cancer ASMR **(A)** and ASDR **(B)** attributable to second-hand smoke in 2021 by sex and EAPC in lung cancer ASMR **(C)** and ASDR **(D)** attributable to second-hand smoke from 1990 to 2021 by sex in 22 regions. ASMR, age-standardized mortality rate; ASDR, age-standardized DALY rate; DALYs, disability-adjusted life-years; EAPC, estimated annual percentage change.

At the level of the GBD region, East Asia bore the heaviest burden (59, 196 deaths and 1.39 million DALYs), accounting for over 60% of lung cancer and DALYs associated with SHS deaths globally in 2021. Meanwhile, East Asia had the highest ASMR (2.75 per 100,000) and ASDR (62.42 per 100,000), followed by Central Europe, North Africa, and Middle East (**Table 1**, **Figures 2A, B**). Between 1990 to 2021, high-income North America, Australasia, Central Latin America, Andean Latin America, and Western Europe experienced a faster decrease in ASMR and ASDR, with EAPCs ranking among the top five. In contrast, Oceania was the only region revealing an increase in ASMR and ASDR, with EAPCs of 0.3 (95% CI: 0.23 to 0.38) and 0.29 (95% CI: 0.21 to 0.37), respectively. Western Sub-Saharan Africa displayed no significant mortality trend in ASMR, with EAPCs of -0.05 (95% CI: -0.13 to 0.03). Although most regions exhibited a downward trend in ASMR and ASDR for both sexes, East Asia showed an increasing trend in both ASMR and ASDR for men, while Oceania and South Asia experienced an increase in both ASMR and ASDR for women (**Table 1**, **Figures 2C, D**).

**Figure 3:**
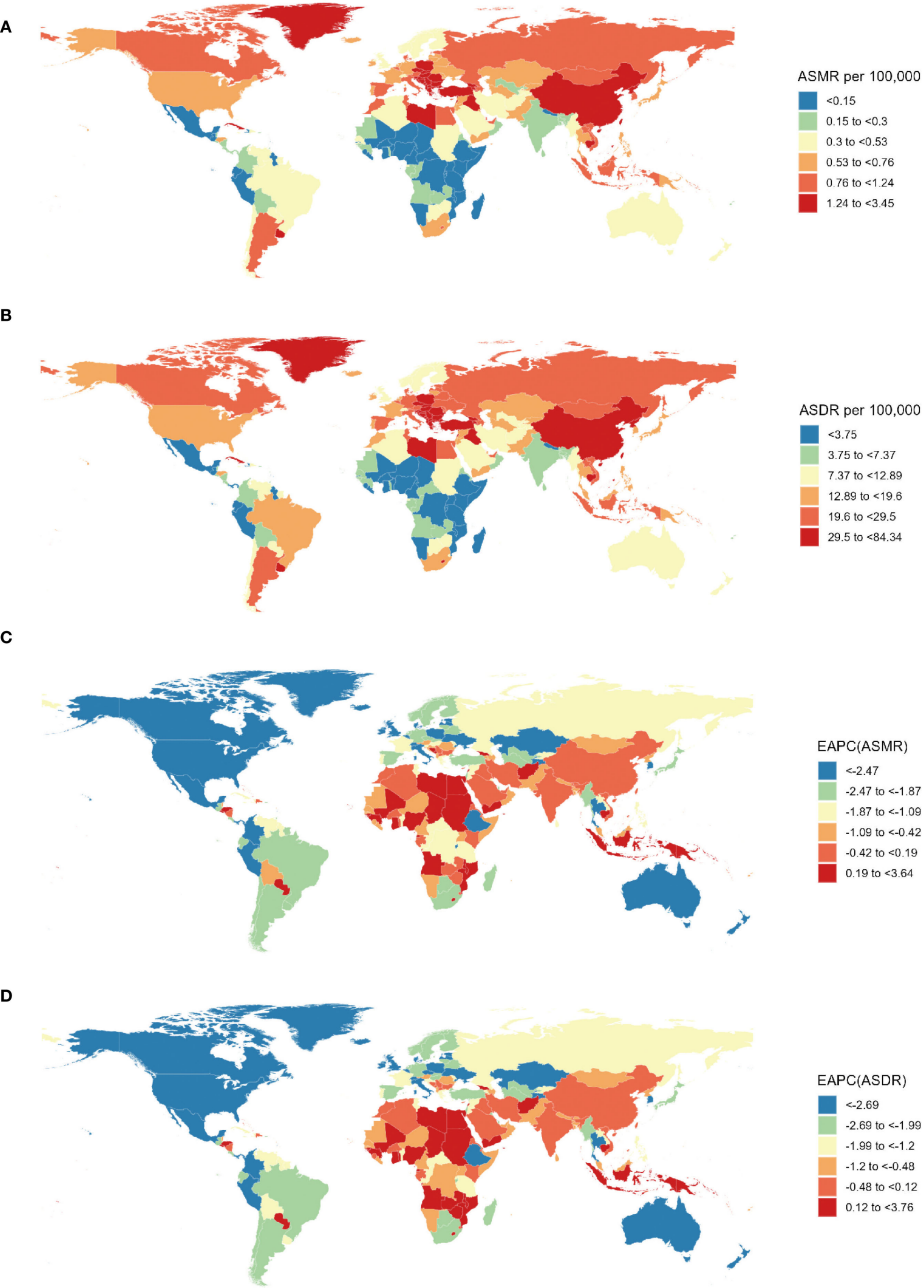
Spatial distribution of lung cancer ASMR **(A)** and ASDR **(B)** and EAPC of lung cancer ASMR **(C)** and ASDR **(B)** attributable to second-hand smoke from 1990 to 2021. ASMR, age-standardized mortality rate; ASDR, age-standardized DALY rate; DALYs, disability-adjusted life-years; EAPC, estimated annual percentage change.

From a national perspective, the People’s Republic of China, the United States of America (USA), and the Republic of India stood out as the top three countries in the number of lung cancer deaths and DALYs related to SHS in 2021 (**Supplementary Table S1**). In 2021, Montenegro, the People’s Republic of China, and North Macedonia ranked as the top three in ASMR and ASDR (**Supplementary Table S1**, **Figures 3A, B**). However, the fastest decrease in ASMR (EAPC=-4.63, 95% CI: -4.83 to -4.42) occurred in Mexico, and the most significant decline in ASDR (EAPC=-4.79, 95% CI: -4.86 to -4.73) was observed in the United Kingdom (UK) (**Supplementary Table S1**, **Figures 3C, D**).

### 3.3 Age and gender variations in global lung cancer burden attributable to second-hand smoke

**Figure 4:**
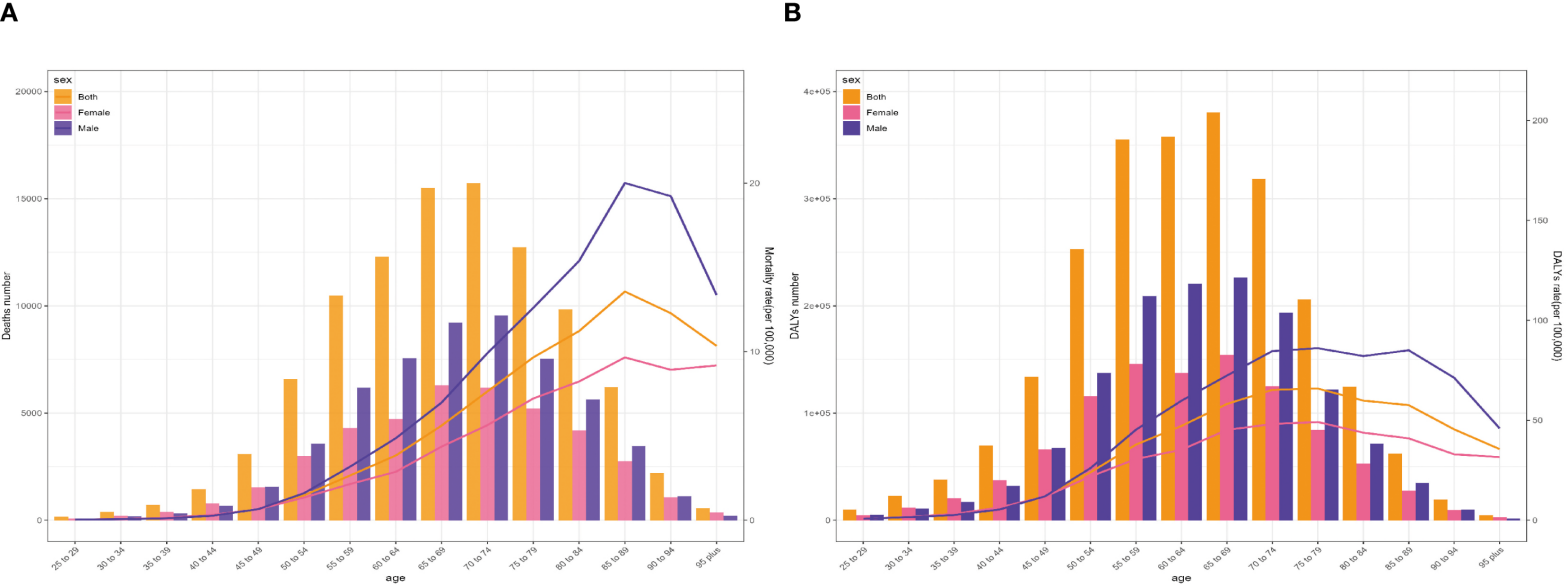
Age-specific numbers and rates of global lung cancer deaths **(A)** and DALYs **(B)** attributable to second-hand smoke by sex in 2021. DALYs, disability-adjusted life-years.

The global age-specific number and rate of deaths and DALYs in 2021 are shown in the diagram of **Figure 4**. The age-specific number of lung cancer deaths reveals an inverted V-shaped distribution pattern with age, culminating in the 70–74 age group. Most deaths were distributed in the 55–79 age bracket, with a higher number in men compared with women. Concerning the age-specific mortality rate related to SHS, the trend exhibited a simultaneous rise and subsequent decline in both men and women across diverse age brackets. The finding demonstrated the crucial role of age as an influencing factor in the impact of SHS on the global lung cancer burden. The mortality rate among individuals increased with age until it peaked in the 85–89 age group, regardless of gender. The mortality ratio of men was substantially higher in comparison with that of women. In contrast, the gender gap in lung cancer mortality rate due to SHS was notably discernible for individuals among the 85–89 age group (**Figure 4A**).

The age-specific number of lung cancer DALYs attributable to SHS manifested a generally similar pattern to the number of deaths. Nevertheless, its highest point occurred in people aged 75–79 years. Correspondingly, the changing pattern of age-specific DALY rate was comparable to the mortality rate. The most apparent sex disparity in lung cancer DALY rate due to SHS was observed among individuals aged 85–89 years (**Figure 4B**).

**Figure 5:**
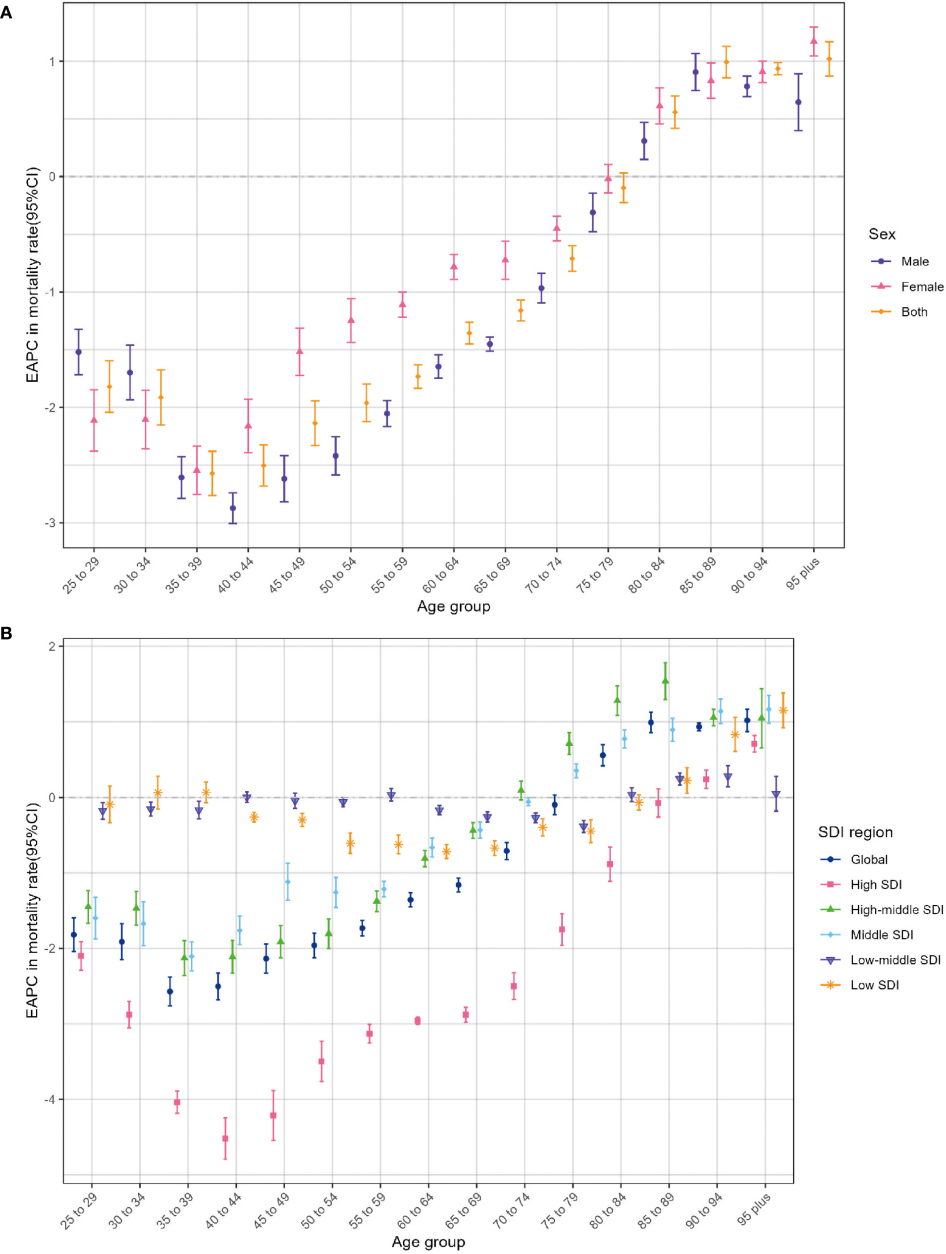
Age distribution of lung cancer EAPC in age-specific mortality rate attributable to second-hand smoke by sex **(A)** and SDI region **(B)** from 1990 to 2021. EAPC, estimated annual percentage change; SDI, sociodemographic index.

Globally, there was a consistent decline below 80 years old and a steady rise above 80 years in age-specific mortality rate among individuals from 1990 to 2021. Specifically, the steepest descent occurred in the 35–39 age group, while the most rapid ascent existed among individuals aged 95 and above. During this period, a roughly consistent pattern of change in mortality rate was observed among men and women across all age brackets. Moreover, it is noteworthy that the age-specific mortality rate among men revealed the sharpest decline in the 40–44 age bracket and witnessed the most apparent increase in the 85–89 age group (**Figure 5A**). It is worth noting that the mortality rates across different age groups remained relatively stable in the low-middle SDI and low SDI regions, with the EAPCs approaching zero (**Figure 5B**). A resembling pattern of variation was likewise observed on the EAPCs of the age-specific DALY ratio (**Supplementary Figures S3A, B**).

### 3.4 Lung cancer burden attributable to second-hand smoke associated with SDI

**Figure 6:**
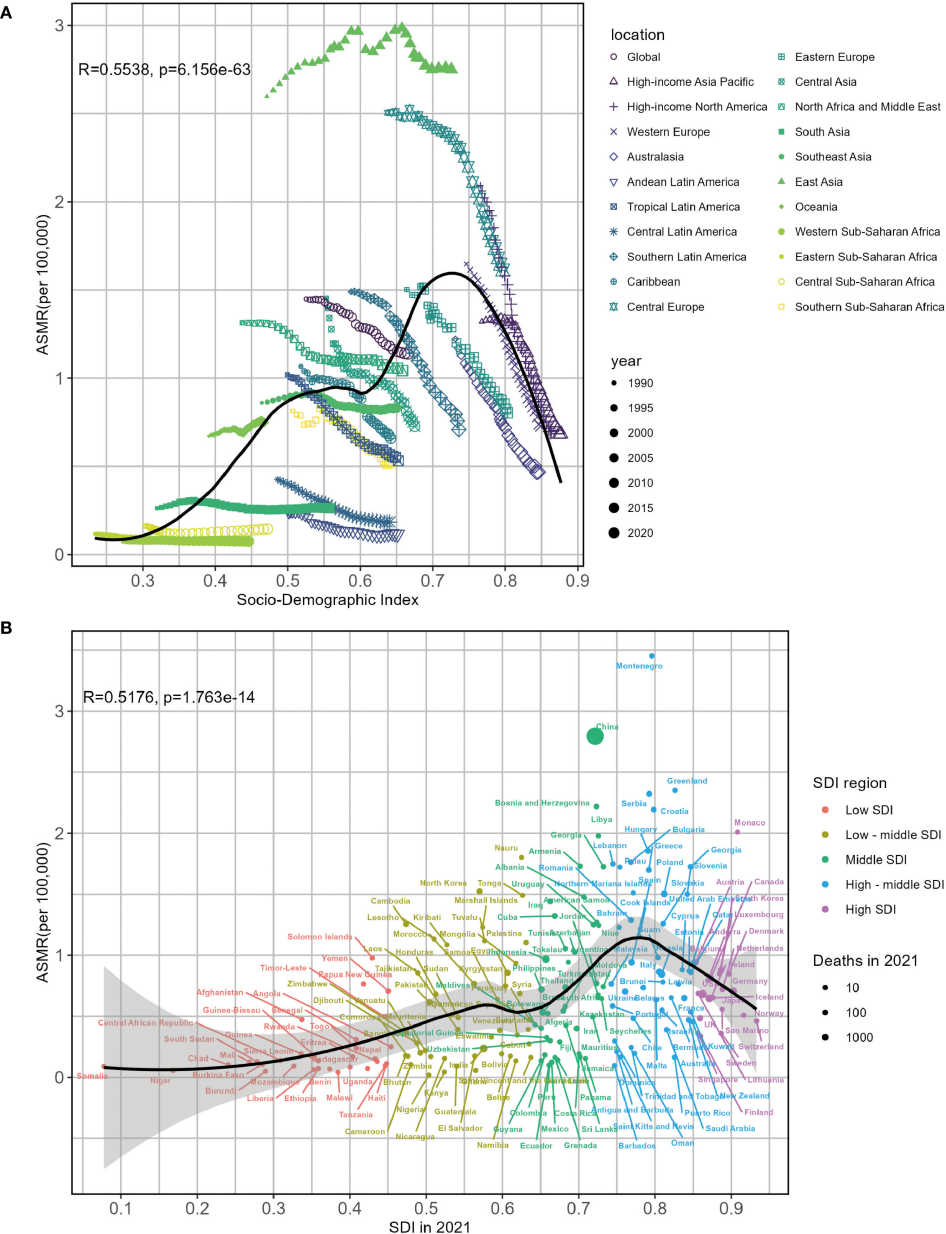
Associations between lung cancer ASMR attributable to second-hand smoke and SDI by 22 regions **(A)** and 204 countries **(B)** in 2021. ASMR, age-standardized mortality rate; SDI, sociodemographic index.

**Figure 6** illustrates the correlations between observed and predictive ASMR of lung cancer linked to SHS and SDI at the regional and national levels, showing a positive relationship. From a regional perspective, regions with middle and high-middle SDI suffered a heavier burden of lung cancer related to SHS. Furthermore, East Asia, North Africa and Middle East, Central Asia, Central Europe, and high-income Asia Pacific had higher observed ASMR than anticipation based on their SDIs from 1990 to 2021. The high SDI regions, such as Western Europe and high-income North America, closely tracked the expected trends over the study period (**Figure 6A**). Numerous countries, such as Montenegro and the People’s Republic of China, showed considerably higher observed rates than foreseen. In contrast, countries including Barbados and the Federation of Saint Kitts and Nevis bore lower burdens than anticipated on account of their SDI values (**Figure 6B**). Regarding EAPC, the EAPC on ASMR displayed a negative association with SDI (*R* = - 0.5024, *P* < 0.0001), especially in countries with SDI belonging to 0.55–0.65 and 0.75–0.9 (**Supplementary Figure S4A**). Similar ASDR patterns were likewise revealed in **Supplementary Figures S4B**, **S5A, B**.

## 4 Discussion

This study exploited the data from the GBD 2021 to thoroughly estimate the spatiotemporal trends in deaths and DALYs of lung cancer attributable to SHS in 204 countries and territories. The global number of deaths and DALYs of SHS-related lung cancer increased significantly by 69.9% and 47.3%, respectively, with ASMR and ASDR decreasing conversely during the past 32 years. The burden of lung cancer induced by SHS is distributed inequitably across the world, with the highest ASMR and ASDR existing in East Asia, Central Europe, and North Africa and Middle East, whereas high-income North America, Australasia, and Central Latin America underwent the most substantial declines from 1990 to 2021. In the SDI district, high-middle SDI regions exhibited the most significant number of lung cancer deaths and DALYs related to SHS. The ASMR or ASDR revealed an approximatively reverse W-shaped relationship with SDI, with the gravest burden noted in the middle and high-middle SDI regions. On the contrary, high SDI regions demonstrated the steepest decline in the ASMR and ASDR of SHS-related lung cancer from 1990 to 2021. Furthermore, men and individuals in the 55–79 age group suffered from a more serious burden of lung cancer attributable to SHS from a demographic perspective. The sex divergence was more evident in the geriatric group.

The latest analysis from GBD 2021 emphasizes a disturbing global trend: the incidence and death numbers of tracheal, bronchus, and lung (TBL) cancer are continuously escalating at a relatively rapid rate across all genders and age groups worldwide. In 2021, it is estimated that approximately 5.2% of total TBL cancer was caused by SHS, making SHS the fourth principal cause of TBL cancer, ranking only behind smoking (59.5%), ambient particulate matter pollution (15.0%), and occupational asbestos exposure (7.2%) (15). SHS encompasses 172 compounds, containing 11 carcinogens identified by the International Agency for Research on Cancer (IARC) (16, 17). Currently, evidence regarding the association between SHS and TBL cancers, especially lung cancer, has been accumulating. Experiments *in vivo* and *in vitro* manifested that tobacco smoke contributed to remarkable alterations in DNA methylation and hydroxymethylation, gene expression profiles, and protein abundance to produce an inflammatory environment and promote lung cancer cell growth (8, 18–20). A meta-analysis revealed that exposure to SHS among non-smokers increased the risk of lung cancer by over 20%, and this risk was closely related to the duration, intensity, and number of pack-years of SHS exposure (6). Hence, with the rapid development of the social economy, individuals devote increasing attention to the impact of SHS on health. In 2005, the WHO issued the Framework Convention on Tobacco Control (FCTC), guiding authorities and civil society to control exposure to SHS based on a substantial body of evidence (21). Numerous countries have legislated for national smoke-free policies covering workplaces and public districts, causing a reduction in the mortality of individuals exposed to SHS (22). Thus, a remarkable decline has occurred in the ASMR and ASDR of lung cancer attributable to SHS exposure. However, with the rapid increase in population and the transformation toward an aging population structure, the number of lung cancer deaths and DALYs attributable to SHS are still increasing, imposing a severe disease burden. Despite more than a decade of tobacco control efforts since the approval of the FCTC, exposure to SHS remains a crucial public health issue. Therefore, the world needs to make greater efforts in formulating more comprehensive smoke-free policies, ensuring the implementation of these policies, and protecting the public from the harm of tobacco smoke. It is worth noting that the coronavirus disease 2019 (COVID-19) pandemic during 2020–2021 disrupted healthcare systems and affected lung cancer screening considerably owing to the lockdown, which limited access among high-risk smokers and enhanced non-smokers engagement (23). It may be the reason why the decreasing trend of ASMR and ASDR slowed down during 2020–2021 among regions.

At the SDI level, the highest ASMR and ASDR occurred in high-middle SDI regions, while the levels of ASMR and ASDR in higher SDI regions were significantly lower. These geographical disparities stem from varying degrees of socio-economic level development, which are the decisive factors for the health outcomes of different populations. The differences in the burden of lung cancer caused by SHS among regions with different SDI levels indicate social–spatial inequalities in aspects such as the prevention of lung cancer, healthcare, and the control of environmental tobacco smoke. In the high-middle SDI regions, the widespread use of tobacco, the public with a deficiency in awareness of tobacco hazards, insufficient legislative supervision by the government, and the particularly potent interest groups within the tobacco industry led to a large amount of SHS exposure among non-smokers, contributing to the occurrence and development of lung cancer and premature deaths (24–26). A study focusing on Central European countries revealed that despite smoke-free laws, there were still obstacles to the implementation of smoke-free areas, such as a lack of resources, a highly competitive business environment, and insufficient social support (27). It is worth noting that some countries with a high SDI have successfully enforced comprehensive tobacco control programs, including publicity and education, increasing tobacco taxes, and formulating tobacco control regulations (28–30). These measures effectively protect the public from exposure to SHS, thus preventing deaths caused by SHS exposure in public places, workplaces, or at home. In addition, advances in the screening and treatment of lung cancer have also contributed to the increase in lung cancer survival rates in high SDI regions. The National Lung Screening Trial conducted in the United States demonstrated that, compared with screening using chest radiographs, the annual implementation of low-dose computed tomography (LDCT) screening could significantly reduce the mortality rate of individuals at a high risk of lung cancer by 20% (31).

Currently, the USA and some countries in Europe with high SDI have identified or implemented optimized screening strategies to improve the survival rate of lung cancer (32–35). However, in low SDI regions, it is challenging to implement lung cancer screening and new treatment methods due to the shortage of medical resources (36). Interestingly, compared with the middle and high SDI regions, low SDI regions underwent a lighter burden of lung cancer associated with SHS. It might be due to the limited healthcare and monitoring systems, which led to underreporting of data on lung cancer and second-hand smoke exposure, or the relatively small number of relevant studies (37). Therefore, more profound research needs to be conducted to investigate the disease burden of lung cancer induced by SHS in the low SDI regions.

The disease burden of lung cancer caused by SHS varies among different countries. Our research indicated that at the national level, Montenegro, the People’s Republic of China, and North Macedonia suffered the heaviest disease burden of lung cancer caused by SHS in 2021. In the Balkan countries, including Montenegro and North Macedonia, tobacco production accounts for 78% of the total cash crop production, an essential source of household income for the people in this region (38). High subsidies for tobacco cultivation from the governments and the widespread prevalence of tobacco tax evasion (39, 40) pose great obstacles to tobacco control and the reduction of second-hand smoke exposure in these countries. China is one of the countries with the highest tobacco usage rates in the world, and more than 740 million non-smokers are exposed to second-hand smoke. Despite the support of national legislation, the increased public awareness of the hazards of tobacco exposure, and the changes in social habits, tobacco control in China remains quite challenging due to the interference of the tobacco industry (24). In recent years, Mexico experiences the steepest decline in the ASMR for lung cancer attributed to SHS. As the primary country among the Americas to ratify the FCTC in 2004, Mexico introduced a new anti-tobacco legislation in 2023, which prohibits smoking in all public places, thus providing strong protection for non-smokers from the harm of SHS (40). Meanwhile, the United Kingdom witnessed the fastest declining trend of the ASDR, which was attributable to its successful public health policies and advanced national healthcare system (41).

Taking into account the gender differences, the ASMR and ASDR of lung cancer attributable to SHS declined more rapidly among men than among women. Women are more significantly affected by exposure to SHS compared with men. Research has revealed that, at equivalent smoking levels, women exhibit a higher susceptibility to lung cancer than men, attributed to their enhanced sensitivity to tobacco carcinogens (42). Moreover, mutations in the tumor suppressor gene P53 and the proto-oncogene K-RAS are more prevalent in women, posing a latent threat to their health (43). It is noteworthy that among non-smoker patients diagnosed with lung cancer, 79% of women claimed to be exposed to second-hand smoke, mainly influenced by their smoking partners at home or exposure in the workplace (44). Recent research has further demonstrated that exposure to tobacco smoke disrupts the circadian rhythm in a gender-specific fashion manner and downregulates the tumor suppressor factors in female cRaf transgenic mice, thus promoting the growth of lung cancer tumors (45). Although women exhibit a greater susceptibility to tobacco exposure in comparison with men, the burden of lung cancer attributable to SHS is more substantial in men. Compared with women, men exhibit a significantly higher level of exposure to SHS within the workplace environment (46, 47). Additionally, non-smokers, as defined by GBD 2021, include occasional smokers and former smokers, increasing the proportion of men within the scope of non-smokers, which may be one of the reasons for the heavier burden of lung cancer among men who report not smoking.

In recent years, lung cancer mortality and DALYs ascribable to SHS have manifested an overall ascendant tendency subsequent to the age of 25. However, this trend reverses at 85 years of age. Individuals in the 55–79 age stratum bore the most substantial burden. Multiple meta-analyses (6, 48) have detected an intimate connection between lung cancer and the duration of SHS exposure. As the duration of SHS exposure lengthens, the probability of developing lung cancer escalates. Long-term exposure to SHS probably elicits chronic lung inflammation, giving rise to epigenetic alterations of diverse magnitudes (19). Research indicated that fewer than half of smokers could live up to 85 years old, which accounts for the alleviation of the SHS-related lung cancer burden among individuals over 85. Moreover, our research noted that the age-specific mortality rate experienced the steepest decline within the 35–40 age cohort, which likely reflects the enhancement of contemporary health consciousness, encompassing efforts such as curtailing male smoking prevalence and shielding pregnant women and young children from SHS. In the future, it is necessary to offer more training courses on maternal and perinatal health to enhance male smokers’ awareness of the hazards of second-hand smoke during pregnancy.

In the whole world, especially in low to middle-high SDI regions, multiple strategies ought to be embraced to mitigate the burden of lung cancer caused by SHS. Firstly, governments should formulate and implement tobacco control policies and smoke-free laws. Secondly, concerted efforts should be made to advocate for an upward adjustment in the price of tobacco products and an augmentation of taxes levied on tobacco manufacturers. At present, the tobacco industry persists as the principal impediment to tobacco control and constitutes a substantial threat to public health. The renowned British anti-smoking organization Action on Smoking and Health (ASH) has emphasized the importance of restricting the development of the tobacco industry and intensifying its supervision of it (49). Thirdly, the hazards of SHS exposure should be actively publicized to raise public health awareness. Fourthly, countries relying predominantly on tobacco production as a major economic pillar should endeavor to explore and identify economically sustainable tobacco substitutes to reduce tobacco cultivation (38). Finally, research funds should be provided for developing countries to provide strong evidence for the negative impacts of tobacco consumption.

To our knowledge, this study is the most current and comprehensive analysis examining the temporal and spatial patterns for the burden of lung cancer attributable to SHS, concentrating on the variations across years, genders, geographic locations, age groups, and SDI categories over the past 32 years. The latest data source, GBD 2021, was utilized for systematic analysis. Despite this, there are still several persisting limitations to consider in this study. First, complete and accurate data about all countries were not encompassed, especially data from low SDI regions such as Sub-Saharan Africa. Data in these regions were usually estimated through mathematical modeling, resulting in inaccurate statistics. Accordingly, it is imperative to exercise prudence when interpreting the outcomes, especially in regions or countries with broader 95% UIs. Second, the GBD database did not take into account the hazards of e-cigarette exposure. Currently, smoke-free laws do not clearly define e-cigarettes, which most e-cigarette users can use in smoke-free areas (50). It is imperative to conduct an in-depth analysis of the lung cancer burden caused by e-cigarette-related SHS. Such an analysis will assist the government in formulating well-defined policies. Third, the available SHS data mainly hinge on self-reporting, making it susceptible to recall and selection bias. Cotinine, as the primary metabolite of nicotine in the body, is quantitatively measured in blood, urine, and saliva samples. It reflects the intake of tobacco smoke more accurately and has been revealed to be associated with the risk of lung cancer in previous studies, which is lacking in the GBD study (51, 52). Notwithstanding the extensive efforts exerted by the GBD collaborators, the potential for underreporting or misclassifying SHS exposure persists across diverse GBD regions and countries. A study manifested that surveys on children’s exposure to SHS at home were based on self-reports from parents or caregivers, which might lead to potential misclassification (5). Since smoking around children is considered socially unacceptable, parents may be reluctant to report their smoking behavior. Fourth, SHS is defined variedly in different studies. SHS was not separated from former active smokers and occasional smokers in the GBD comparative-risk framework, inflating the male burden and inducing residual confounding bias. Fifth, certain confounding factors, including the impacts of genetic factors, behavior, and dietary patterns, have not been taken into account. These factors may confound the observed relationship between SHS and lung cancer. Finally, there are significant disparities in lung cancer diagnostic methods and tumor registrations across countries or regions with different SDI levels. It is a requisite that cancer registries in each country annually amass information featuring explicit pathological diagnoses to mitigate such concerns.

## 5 Conclusion

During the past 32 years, the global burden of lung cancer attributable to SHS has revealed a declining tendency, concomitant with a decline in SHS exposure. Significant gender, age, and regional epidemiological disparities persist. The burden of lung cancer attributable to SHS is more substantial among men within the 55–79 age bracket and in the high-middle SDI regions. These findings identify the most affected demographics and regions, providing valuable insights for policymakers in the health and environmental sectors.

## Data Availability

The original contributions presented in the study are included in the article/**Supplementary Material**. Further inquiries can be directed to the corresponding author.
