# Supplementary material for: Global, regional, and national burden of lung cancer attributable to second-hand smoke from 1990 to 2021: a systematic analysis based on the Global Burden of Disease Study 2021
Source: Front Oncol. 2025 Oct 7;15:1609230. doi: 10.3389/fonc.2025.1609230 (PMC12537378; doi:10.3389/fonc.2025.1609230)
Supplement: Supplementary file 1 [file DataSheet1.docx]

Table S1 Global lung cancer burden attributable to second-hand smoke in 1990 and 2021, and EAPCs of both ASMR and ASDR in 204 countries and territories from 1990 to 2021.

|  | **1990** | | | | **2021** | | | | **EAPC(1990-2021)** | | |
| --- | --- | --- | --- | --- | --- | --- | --- | --- | --- | --- | --- |
| **location** | **Mortality cases (95% UI)** | **ASMR (95% UI)** | **DALYs (95% UI)** | **ASDR (95% UI)** | **Mortality cases (95% UI)** | **ASMR (95% UI)** | **DALYs (95% UI)** | **ASDR (95% UI)** | **ASMR(95% UI)** | | **ASDR(95% UI)** |
| **Afghanistan** | 30 (3-82) | 0.44 (0.04-1.16) | 854 (78-2,280) | 11.52 (1.05-30.4) | 46 (4-111) | 0.47 (0.05-1.1) | 1,477 (142-3,554) | 12.34 (1.19-29.4) | 0.64 (0.48 to 0.79) | 0.6 (0.45 to 0.75) | |
| **Albania** | 40 (5-80) | 1.98 (0.24-3.93) | 1,077 (125-2,141) | 49.24 (5.83-97.94) | 65 (7-134) | 1.48 (0.16-3.06) | 1,519 (168-3,145) | 35.61 (3.92-73.67) | -0.86 (-1.13 to -0.59) | -0.94 (-1.21 to -0.66) | |
| **Algeria** | 70 (9-139) | 0.63 (0.08-1.24) | 1,890 (228-3,715) | 14.9 (1.8-29.38) | 176 (22-354) | 0.52 (0.07-1.06) | 4,632 (572-9,417) | 12.33 (1.54-24.96) | -0.33 (-0.57 to -0.08) | -0.47 (-0.65 to -0.29) | |
| **American Samoa** | 0 (0-1) | 1.39 (0.14-3.42) | 8 (1-18) | 32.13 (3.43-76.92) | 1 (0-1) | 1.27 (0.12-3.12) | 15 (1-36) | 29.55 (2.8-72.19) | -0.24 (-0.3 to -0.19) | -0.23 (-0.29 to -0.17) | |
| **Andorra** | 1 (0-3) | 1.93 (0.22-4.59) | 31 (4-74) | 53.21 (6.05-125.37) | 1 (0-3) | 0.8 (0.09-1.77) | 33 (4-73) | 21.46 (2.31-47.57) | -2.65 (-2.88 to -2.42) | -2.7 (-2.9 to -2.5) | |
| **Angola** | 9 (1-22) | 0.21 (0.03-0.5) | 301 (38-715) | 6.2 (0.77-14.7) | 32 (4-71) | 0.25 (0.03-0.55) | 1,051 (126-2,326) | 7.08 (0.85-15.55) | 0.84 (0.49 to 1.19) | 0.69 (0.34 to 1.04) | |
| **Antigua and Barbuda** | 0 (0-0) | 0.31 (0.04-0.68) | 4 (1-9) | 7.79 (1.02-16.96) | 0 (0-1) | 0.21 (0.02-0.49) | 5 (1-13) | 4.92 (0.47-11.67) | -1.32 (-1.6 to -1.04) | -1.47 (-1.75 to -1.19) | |
| **Argentina** | 524 (60-1,124) | 1.62 (0.19-3.46) | 1,5038 (1,741-32,031) | 46.16 (5.36-98.29) | 423 (53-989) | 0.77 (0.1-1.79) | 11,013 (1,420-25,282) | 20.58 (2.68-47.34) | -2.22 (-2.5 to -1.94) | -2.46 (-2.77 to -2.16) | |
| **Armenia** | 68 (8-134) | 2.36 (0.27-4.67) | 2,021 (232-4,004) | 66.52 (7.59-131.58) | 76 (10-145) | 1.73 (0.23-3.28) | 1,836 (251-3,532) | 42.21 (5.8-80.63) | -0.74 (-1 to -0.47) | -1.28 (-1.52 to -1.04) | |
| **Australia** | 240 (23-517) | 1.24 (0.12-2.65) | 6,377 (621-13,629) | 33.6 (3.32-71.78) | 205 (21-453) | 0.47 (0.05-1.03) | 4,870 (491-10,619) | 12.06 (1.22-25.89) | -3.07 (-3.19 to -2.95) | -3.24 (-3.34 to -3.14) | |
| **Austria** | 131 (14-279) | 1.19 (0.13-2.54) | 3,570 (398-7,572) | 34.49 (3.89-72.88) | 159 (22-311) | 0.95 (0.13-1.85) | 4,004 (550-7,801) | 25.57 (3.51-49.78) | -0.45 (-0.76 to -0.14) | -0.68 (-1.03 to -0.32) | |
| **Azerbaijan** | 78 (10-156) | 1.47 (0.18-2.97) | 2,367 (291-4,739) | 42.24 (5.23-85.34) | 110 (14-221) | 1.03 (0.13-2.03) | 3,203 (386-6,428) | 27.26 (3.4-54.54) | -0.71 (-1 to -0.42) | -1.08 (-1.35 to -0.8) | |
| **Bahamas** | 1 (0-2) | 0.45 (0.05-0.99) | 20 (2-45) | 12.27 (1.5-27.19) | 2 (0-3) | 0.37 (0.04-0.84) | 42 (4-93) | 9.65 (0.94-21.26) | -0.23 (-0.37 to -0.09) | -0.39 (-0.53 to -0.25) | |
| **Bahrain** | 3 (0-7) | 2.25 (0.28-4.44) | 95 (12-189) | 50.81 (6.39-99.89) | 9 (1-18) | 1.23 (0.15-2.47) | 261 (32-511) | 25.94 (3.26-51.59) | -2.44 (-2.74 to -2.14) | -2.7 (-2.96 to -2.43) | |
| **Bangladesh** | 118 (15-263) | 0.25 (0.03-0.56) | 3,343 (432-7,451) | 6.72 (0.87-14.99) | 273 (29-632) | 0.2 (0.02-0.46) | 7,234 (761-16,578) | 5.02 (0.53-11.49) | -1.03 (-1.28 to -0.79) | -1.16 (-1.41 to -0.91) | |
| **Barbados** | 1 (0-1) | 0.18 (0.02-0.41) | 12 (1-27) | 4.51 (0.48-10.52) | 1 (0-1) | 0.1 (0.01-0.25) | 12 (1-32) | 2.43 (0.22-6.29) | -2.12 (-2.41 to -1.83) | -2.13 (-2.4 to -1.85) | |
| **Belarus** | 162 (16-340) | 1.23 (0.12-2.55) | 4,884 (490-10,226) | 37.09 (3.71-76.96) | 118 (12-249) | 0.73 (0.07-1.55) | 3,258 (320-6,900) | 20.96 (2.08-44.38) | -2.17 (-2.39 to -1.95) | -2.39 (-2.61 to -2.17) | |
| **Belgium** | 334 (43-667) | 2.28 (0.29-4.51) | 8,892 (1,158-17,643) | 63.84 (8.38-125.97) | 204 (24-423) | 0.94 (0.11-1.94) | 4,943 (581-10,050) | 25.07 (2.96-49.99) | -2.6 (-2.81 to -2.39) | -2.79 (-3.04 to -2.53) | |
| **Belize** | 0 (0-1) | 0.24 (0.03-0.58) | 6 (1-14) | 6.11 (0.64-14.53) | 1 (0-1) | 0.17 (0.02-0.39) | 15 (2-33) | 4.52 (0.5-10.17) | -0.97 (-1.29 to -0.66) | -0.88 (-1.16 to -0.6) | |
| **Benin** | 2 (0-5) | 0.1 (0.01-0.23) | 53 (6-123) | 2.57 (0.29-5.99) | 3 (0-8) | 0.07 (0.01-0.16) | 96 (9-219) | 1.7 (0.17-3.87) | -1.34 (-1.4 to -1.27) | -1.44 (-1.51 to -1.36) | |
| **Bermuda** | 1 (0-2) | 1.1 (0.1-2.56) | 17 (1-38) | 26.3 (2.34-60.5) | 1 (0-2) | 0.65 (0.05-1.49) | 19 (2-45) | 15.17 (1.21-34.67) | -1.48 (-1.64 to -1.32) | -1.63 (-1.8 to -1.46) | |
| **Bhutan** | 0 (0-1) | 0.19 (0.02-0.44) | 15 (2-34) | 5.21 (0.61-12.03) | 1 (0-3) | 0.18 (0.02-0.48) | 29 (3-77) | 4.56 (0.52-12.21) | -0.26 (-0.44 to -0.07) | -0.51 (-0.69 to -0.33) | |
| **Bolivia (Plurinational State of)** | 9 (1-18) | 0.28 (0.03-0.57) | 244 (29-494) | 7.11 (0.84-14.4) | 17 (2-37) | 0.19 (0.02-0.42) | 449 (49-958) | 4.68 (0.51-9.99) | -0.99 (-1.29 to -0.68) | -1.21 (-1.52 to -0.9) | |
| **Bosnia and Herzegovina** | 95 (10-186) | 2.16 (0.23-4.24) | 2,791 (302-5,496) | 59.75 (6.49-116.94) | 139 (18-287) | 2.22 (0.28-4.57) | 3,429 (448-7,109) | 56.58 (7.39-118.43) | 0.37 (0.04 to 0.71) | 0.07 (-0.26 to 0.4) | |
| **Botswana** | 4 (0-9) | 0.68 (0.07-1.58) | 112 (11-259) | 18.12 (1.77-42.31) | 6 (1-15) | 0.43 (0.05-0.99) | 184 (21-425) | 11.12 (1.32-25.73) | -1.89 (-2.26 to -1.52) | -2.05 (-2.45 to -1.65) | |
| **Brazil** | 907 (110-1,742) | 1.03 (0.13-1.99) | 25,134 (3,072-48,037) | 26.4 (3.22-50.53) | 1,342 (159-2,687) | 0.53 (0.06-1.07) | 33,692 (4,046-67,710) | 13.12 (1.57-26.34) | -2.34 (-2.45 to -2.24) | -2.48 (-2.58 to -2.38) | |
| **Brunei Darussalam** | 2 (0-4) | 1.95 (0.26-3.82) | 54 (7-105) | 47.74 (6.32-93.39) | 3 (0-6) | 0.78 (0.07-1.71) | 76 (7-166) | 18.97 (1.75-41.57) | -2.53 (-2.78 to -2.28) | -2.63 (-2.89 to -2.37) | |
| **Bulgaria** | 264 (32-499) | 2.12 (0.26-4.01) | 7,821 (963-14,723) | 63.16 (7.88-118.94) | 232 (25-499) | 1.76 (0.19-3.8) | 6,150 (669-13,228) | 50.42 (5.49-108.65) | -0.26 (-0.45 to -0.06) | -0.43 (-0.63 to -0.23) | |
| **Burkina Faso** | 5 (1-10) | 0.11 (0.01-0.24) | 131 (14-276) | 2.85 (0.32-5.97) | 10 (1-22) | 0.11 (0.01-0.25) | 258 (28-579) | 2.61 (0.29-5.95) | -0.06 (-0.3 to 0.17) | -0.15 (-0.4 to 0.11) | |
| **Burundi** | 2 (0-5) | 0.1 (0.01-0.22) | 75 (8-161) | 2.93 (0.32-6.29) | 3 (0-6) | 0.05 (0.01-0.11) | 87 (10-199) | 1.43 (0.16-3.27) | -2.65 (-3.05 to -2.25) | -2.69 (-3.09 to -2.29) | |
| **Cabo Verde** | 1 (0-1) | 0.23 (0.02-0.55) | 13 (1-31) | 5.7 (0.56-14.14) | 1 (0-3) | 0.28 (0.03-0.64) | 31 (3-69) | 6.61 (0.67-14.9) | 0.24 (-0.18 to 0.66) | 0.09 (-0.27 to 0.44) | |
| **Cambodia** | 39 (4-88) | 0.9 (0.08-2.03) | 1,108 (107-2,505) | 22.72 (2.15-51.32) | 149 (14-311) | 1.25 (0.12-2.62) | 4,056 (383-8,497) | 30.48 (2.86-64.13) | 0.97 (0.82 to 1.11) | 0.82 (0.67 to 0.97) | |
| **Cameroon** | 6 (1-14) | 0.13 (0.01-0.32) | 160 (17-384) | 3.33 (0.36-7.96) | 13 (1-31) | 0.11 (0.01-0.25) | 366 (35-884) | 2.62 (0.25-6.33) | -0.78 (-0.88 to -0.69) | -0.78 (-0.9 to -0.67) | |
| **Canada** | 842 (90-1,598) | 2.65 (0.28-5.02) | 23,206 (2,487-43,957) | 74.6 (8.03-141.38) | 581 (57-1186) | 0.81 (0.08-1.66) | 13,644 (1,395-27,813) | 20.53 (2.12-41.49) | -3.8 (-3.94 to -3.67) | -4.17 (-4.32 to -4.02) | |
| **Central African Republic** | 2 (0-6) | 0.19 (0.02-0.48) | 76 (8-194) | 5.51 (0.54-14.06) | 3 (0-10) | 0.14 (0.01-0.37) | 116 (10-333) | 4.02 (0.34-11.17) | -1.21 (-1.39 to -1.03) | -1.21 (-1.4 to -1.03) | |
| **Chad** | 2 (0-5) | 0.08 (0.01-0.19) | 60 (7-145) | 2.06 (0.22-4.97) | 6 (1-14) | 0.1 (0.01-0.25) | 168 (19-409) | 2.63 (0.3-6.35) | 0.93 (0.87 to 0.99) | 0.82 (0.75 to 0.89) | |
| **Chile** | 92 (10-201) | 0.93 (0.1-2.02) | 2,361 (259-5,170) | 22.87 (2.51-50.08) | 126 (15-269) | 0.48 (0.06-1.04) | 2,797 (344-5,964) | 10.88 (1.34-23.2) | -2.02 (-2.09 to -1.94) | -2.33 (-2.4 to -2.26) | |
| **China** | 20,758 (2,564-39,187) | 2.63 (0.32-4.96) | 581,552 (72,614-1,097,292) | 65.04 (8.06-122.56) | 58,034 (7,170-109,625) | 2.8 (0.35-5.27) | 1,359,730 (170,188-2,537,368) | 63.32 (7.95-117.85) | 0.16 (0.03 to 0.28) | -0.17 (-0.28 to -0.07) | |
| **Colombia** | 73 (8-149) | 0.43 (0.05-0.88) | 1,954 (221-4,020) | 10.52 (1.18-21.59) | 100 (11-209) | 0.18 (0.02-0.38) | 2,283 (256-4,765) | 4.13 (0.46-8.63) | -3.18 (-3.32 to -3.03) | -3.41 (-3.56 to -3.26) | |
| **Comoros** | 0 (0-1) | 0.2 (0.02-0.44) | 11 (1-25) | 5.24 (0.56-11.54) | 1 (0-2) | 0.17 (0.02-0.4) | 23 (3-53) | 4.4 (0.52-10.03) | -0.73 (-0.84 to -0.63) | -0.9 (-1.04 to -0.76) | |
| **Congo** | 3 (0-8) | 0.3 (0.04-0.72) | 102 (13-244) | 8.48 (1.06-19.94) | 7 (1-17) | 0.24 (0.02-0.59) | 217 (21-531) | 6.59 (0.65-15.89) | -1.11 (-1.4 to -0.83) | -1.22 (-1.52 to -0.92) | |
| **Cook Islands** | 0 (0-0) | 1.55 (0.2-3.52) | 4 (1-10) | 34.98 (4.62-80.7) | 0 (0-1) | 1.16 (0.13-2.85) | 7 (1-17) | 26.05 (2.97-65.36) | -1.17 (-1.31 to -1.02) | -1.13 (-1.26 to -1) | |
| **Costa Rica** | 7 (1-17) | 0.4 (0.04-0.99) | 168 (17-413) | 9.53 (0.95-23.48) | 11 (1-27) | 0.19 (0.02-0.48) | 256 (25-638) | 4.61 (0.45-11.53) | -2.29 (-2.45 to -2.13) | -2.33 (-2.5 to -2.15) | |
| **Coted'Ivoire** | 6 (1-15) | 0.16 (0.02-0.38) | 187 (18-431) | 4.17 (0.4-9.69) | 12 (1-31) | 0.11 (0.01-0.29) | 355 (39-893) | 2.83 (0.31-7.19) | -2.4 (-3.11 to -1.68) | -2.43 (-3.13 to -1.72) | |
| **Croatia** | 226 (31-426) | 3.56 (0.49-6.72) | 6,413 (907-12,028) | 98.07 (13.8-183.92) | 191 (23-400) | 2.19 (0.26-4.59) | 4,520 (530-9,454) | 55.99 (6.57-117.72) | -1.06 (-1.23 to -0.89) | -1.36 (-1.54 to -1.17) | |
| **Cuba** | 207 (25-427) | 2.03 (0.25-4.18) | 4,678 (567-9,708) | 45.52 (5.51-94.24) | 264 (28-576) | 1.32 (0.14-2.88) | 5,772 (606-12,513) | 29.88 (3.15-64.61) | -1.42 (-1.62 to -1.21) | -1.47 (-1.7 to -1.24) | |
| **Cyprus** | 9 (1-18) | 1.15 (0.12-2.31) | 230 (26-449) | 28.62 (3.22-56.7) | 18 (2-36) | 0.88 (0.11-1.76) | 443 (59-889) | 22.27 (2.96-44.82) | -0.26 (-0.5 to -0.02) | -0.19 (-0.43 to 0.06) | |
| **Czechia** | 369 (41-753) | 2.74 (0.31-5.58) | 10,366 (1,159-20,993) | 79.04 (8.88-160.03) | 274 (31-554) | 1.27 (0.15-2.57) | 6,154 (710-12,366) | 30.97 (3.61-61.99) | -2.38 (-2.46 to -2.31) | -2.95 (-3.03 to -2.87) | |
| **Democratic People's Republic of Korea** | 306 (36-621) | 1.92 (0.23-3.9) | 8,901 (1,018-18,049) | 50.04 (5.83-102.18) | 506 (39-1185) | 1.52 (0.12-3.56) | 13,776 (1,112-32,201) | 40.06 (3.22-93.62) | -0.75 (-0.85 to -0.65) | -0.74 (-0.83 to -0.65) | |
| **Democratic Republic of the Congo** | 21 (2-45) | 0.13 (0.01-0.29) | 629 (65-1,340) | 3.54 (0.37-7.62) | 37 (4-89) | 0.1 (0.01-0.24) | 1,147 (121-2,783) | 2.63 (0.27-6.32) | -1.18 (-1.53 to -0.84) | -1.11 (-1.45 to -0.77) | |
| **Denmark** | 209 (23-430) | 2.77 (0.31-5.71) | 5,447 (613-11,180) | 77.17 (8.7-158.48) | 99 (12-206) | 0.85 (0.1-1.76) | 2,196 (272-4,548) | 20.75 (2.58-42.65) | -3.99 (-4.09 to -3.89) | -4.38 (-4.5 to -4.27) | |
| **Djibouti** | 0 (0-1) | 0.27 (0.03-0.62) | 11 (1-25) | 6.82 (0.79-16.13) | 2 (0-3) | 0.28 (0.03-0.58) | 50 (5-102) | 6.87 (0.74-13.99) | 0.16 (0.12 to 0.2) | -0.02 (-0.05 to 0.02) | |
| **Dominica** | 0 (0-1) | 0.38 (0.04-0.94) | 5 (1-13) | 9.12 (1.01-22.18) | 0 (0-1) | 0.3 (0.03-0.75) | 6 (1-16) | 7.3 (0.83-18.07) | -0.79 (-1.05 to -0.53) | -0.69 (-0.95 to -0.42) | |
| **Dominican Republic** | 14 (1-31) | 0.41 (0.04-0.89) | 387 (36-842) | 9.91 (0.91-21.77) | 34 (3-87) | 0.35 (0.03-0.87) | 878 (70-2,242) | 8.58 (0.68-21.93) | -0.24 (-0.58 to 0.1) | -0.21 (-0.48 to 0.06) | |
| **Ecuador** | 10 (1-21) | 0.19 (0.02-0.41) | 263 (31-582) | 4.66 (0.54-10.31) | 16 (2-31) | 0.1 (0.01-0.2) | 383 (50-766) | 2.3 (0.3-4.6) | -2.16 (-2.4 to -1.92) | -2.39 (-2.64 to -2.14) | |
| **Egypt** | 115 (12-222) | 0.4 (0.04-0.77) | 3772 (395-7334) | 11.33 (1.2-22.05) | 536 (59-1118) | 0.86 (0.09-1.81) | 16,066 (1,757-33,367) | 21.9 (2.41-45.84) | 3.41 (2.88 to 3.93) | 2.91 (2.45 to 3.37) | |
| **El Salvador** | 4 (0-9) | 0.12 (0.01-0.29) | 102 (10-238) | 3.27 (0.33-7.69) | 11 (1-25) | 0.17 (0.02-0.41) | 279 (27-652) | 4.6 (0.45-10.76) | 1.18 (0.95 to 1.42) | 1.11 (0.89 to 1.33) | |
| **Equatorial Guinea** | 1 (0-1) | 0.28 (0.03-0.67) | 17 (2-41) | 7.62 (0.79-18.49) | 2 (0-4) | 0.33 (0.04-0.73) | 49 (6-111) | 8.36 (0.95-18.8) | 0.72 (0.59 to 0.86) | 0.45 (0.29 to 0.61) | |
| **Eritrea** | 2 (0-6) | 0.17 (0.02-0.46) | 77 (8-208) | 5.1 (0.53-13.77) | 5 (0-14) | 0.17 (0.02-0.44) | 170 (16-445) | 4.83 (0.45-12.71) | -0.1 (-0.27 to 0.07) | -0.29 (-0.45 to -0.13) | |
| **Estonia** | 40 (4-84) | 1.94 (0.21-4.06) | 1,147 (125-2,396) | 56.06 (6.11-117.42) | 23 (3-49) | 0.87 (0.1-1.88) | 513 (58-1,099) | 21.54 (2.42-46.13) | -2.63 (-2.89 to -2.37) | -3.22 (-3.48 to -2.96) | |
| **Eswatini** | 1 (0-2) | 0.35 (0.04-0.78) | 31 (4-69) | 9.5 (1.11-21.31) | 2 (0-4) | 0.33 (0.04-0.72) | 63 (7-138) | 9.44 (1.13-20.76) | -0.07 (-0.63 to 0.49) | 0.12 (-0.49 to 0.74) | |
| **Ethiopia** | 33 (4-69) | 0.15 (0.02-0.31) | 1,067 (136-2,252) | 4.53 (0.58-9.39) | 32 (4-63) | 0.07 (0.01-0.14) | 982 (125-1,912) | 1.97 (0.25-3.88) | -2.82 (-3.06 to -2.57) | -3.09 (-3.34 to -2.83) | |
| **Fiji** | 1 (0-2) | 0.35 (0.04-0.65) | 30 (4-58) | 8.09 (0.94-15.4) | 2 (0-4) | 0.29 (0.03-0.6) | 53 (6-109) | 6.66 (0.76-13.63) | -0.74 (-1.04 to -0.43) | -0.74 (-1.04 to -0.44) | |
| **Finland** | 53 (6-119) | 0.77 (0.08-1.72) | 1,438 (156-3,210) | 21.61 (2.34-48.05) | 39 (4-91) | 0.34 (0.03-0.77) | 908 (94-2,101) | 8.83 (0.93-20.03) | -2.27 (-2.45 to -2.1) | -2.48 (-2.67 to -2.28) | |
| **France** | 839 (83-1,930) | 1.13 (0.11-2.59) | 24,566 (2,496-56,289) | 34.92 (3.6-79.81) | 764 (80-1,787) | 0.65 (0.07-1.5) | 20,427 (2,148-47,106) | 19.41 (2.04-44.32) | -1.71 (-1.99 to -1.43) | -1.82 (-2.13 to -1.5) | |
| **Gabon** | 2 (0-3) | 0.26 (0.03-0.54) | 44 (5-89) | 7.2 (0.79-14.66) | 3 (0-7) | 0.27 (0.03-0.61) | 90 (9-205) | 7.37 (0.74-16.76) | 0.04 (-0.06 to 0.14) | -0.03 (-0.14 to 0.08) | |
| **Gambia** | 0 (0-1) | 0.11 (0.01-0.26) | 10 (1-25) | 2.65 (0.24-6.67) | 1 (0-2) | 0.09 (0.01-0.23) | 22 (2-58) | 2.14 (0.21-5.64) | -0.95 (-1.2 to -0.7) | -0.98 (-1.23 to -0.73) | |
| **Georgia** | 107 (13-219) | 1.66 (0.2-3.38) | 3,138 (382-6,399) | 47.91 (5.75-97.93) | 102 (13-201) | 1.73 (0.21-3.39) | 2,587 (309-5,125) | 45.41 (5.38-89.6) | 1.62 (1.14 to 2.1) | 1.21 (0.77 to 1.65) | |
| **Germany** | 1,581 (184-3,204) | 1.34 (0.16-2.7) | 45,958 (5,487-92,520) | 40.51 (4.9-81.43) | 1,219 (124-2,624) | 0.71 (0.07-1.52) | 30,896 (3,216-66,680) | 19.72 (2.12-42.48) | -1.98 (-2.04 to -1.93) | -2.25 (-2.3 to -2.21) | |
| **Ghana** | 3 (0-7) | 0.05 (0.01-0.1) | 98 (13-197) | 1.38 (0.19-2.78) | 9 (1-21) | 0.06 (0.01-0.13) | 261 (30-584) | 1.41 (0.16-3.17) | 0.45 (0.33 to 0.57) | 0.29 (0.17 to 0.42) | |
| **Greece** | 392 (50-762) | 2.58 (0.33-5.03) | 9,886 (1,293-19,079) | 66.99 (8.78-129.02) | 373 (34-754) | 1.7 (0.16-3.42) | 8,429 (794-16,968) | 43.15 (4.13-86.94) | -1.42 (-1.59 to -1.25) | -1.44 (-1.62 to -1.25) | |
| **Greenland** | 2 (0-5) | 5.99 (0.57-13.07) | 66 (7-142) | 163.49 (16.03-355.98) | 2 (0-4) | 2.35 (0.24-5.62) | 49 (5-116) | 62.29 (6.26-148.42) | -3.21 (-3.39 to -3.03) | -3.32 (-3.49 to -3.14) | |
| **Grenada** | 0 (0-0) | 0.28 (0.03-0.7) | 5 (1-12) | 7.45 (0.84-18.13) | 0 (0-0) | 0.16 (0.02-0.36) | 5 (1-11) | 4.05 (0.44-9.07) | -1.78 (-2.16 to -1.4) | -1.84 (-2.09 to -1.58) | |
| **Guam** | 1 (0-2) | 1.52 (0.18-3.49) | 27 (3-65) | 34.45 (4.25-80.45) | 2 (0-5) | 0.98 (0.09-2.48) | 55 (5-136) | 25.84 (2.5-64.32) | -0.99 (-1.26 to -0.72) | -0.56 (-0.8 to -0.32) | |
| **Guatemala** | 5 (1-9) | 0.15 (0.02-0.28) | 143 (18-274) | 3.71 (0.46-7.08) | 10 (1-19) | 0.09 (0.01-0.17) | 262 (29-510) | 2.27 (0.25-4.4) | -2.12 (-2.42 to -1.81) | -2.11 (-2.38 to -1.84) | |
| **Guinea** | 5 (1-13) | 0.16 (0.02-0.42) | 144 (14-366) | 4.2 (0.4-10.61) | 11 (1-27) | 0.19 (0.02-0.5) | 307 (25-778) | 5.01 (0.41-12.72) | 0.49 (0.4 to 0.57) | 0.58 (0.48 to 0.68) | |
| **Guinea-Bissau** | 1 (0-1) | 0.13 (0.01-0.32) | 15 (1-39) | 3.45 (0.33-8.93) | 1 (0-3) | 0.18 (0.01-0.44) | 41 (3-98) | 4.75 (0.36-11.58) | 1.72 (1.28 to 2.16) | 1.58 (1.16 to 2) | |
| **Guyana** | 1 (0-2) | 0.22 (0.02-0.53) | 24 (3-56) | 5.84 (0.63-13.79) | 1 (0-2) | 0.14 (0.01-0.34) | 26 (3-64) | 3.66 (0.36-9.09) | -1.49 (-1.69 to -1.3) | -1.36 (-1.57 to -1.15) | |
| **Haiti** | 6 (1-14) | 0.2 (0.02-0.44) | 180 (20-394) | 5.1 (0.57-11.21) | 8 (1-17) | 0.11 (0.01-0.25) | 229 (24-497) | 2.85 (0.3-6.26) | -1.72 (-1.98 to -1.45) | -1.79 (-2.07 to -1.51) | |
| **Honduras** | 9 (1-22) | 0.45 (0.05-1.09) | 254 (26-633) | 11.49 (1.16-28.59) | 42 (4-113) | 0.68 (0.07-1.83) | 1,102 (107-2,991) | 16.51 (1.61-44.63) | 1.57 (1.4 to 1.74) | 1.32 (1.17 to 1.47) | |
| **Hungary** | 444 (59-847) | 3.11 (0.41-5.96) | 13,073 (1,671-25,217) | 94.16 (12.03-182.23) | 337 (35-707) | 1.86 (0.19-3.87) | 8,685 (891-18,199) | 51.32 (5.2-107.55) | -1.83 (-2.12 to -1.54) | -2.17 (-2.52 to -1.81) | |
| **Iceland** | 4 (0-8) | 1.48 (0.17-2.85) | 107 (13-204) | 40.58 (4.8-77.38) | 4 (0-7) | 0.65 (0.08-1.31) | 91 (11-184) | 17.6 (2.1-35.69) | -2.6 (-2.69 to -2.5) | -2.7 (-2.79 to -2.6) | |
| **India** | 950 (124-1,851) | 0.21 (0.03-0.4) | 28,196 (3,678-54,865) | 5.44 (0.71-10.61) | 2,816 (390-5,584) | 0.24 (0.03-0.47) | 78,460 (10,723-154,773) | 6.17 (0.85-12.18) | 0 (-0.2 to 0.2) | -0.01 (-0.2 to 0.19) | |
| **Indonesia** | 596 (67-1,232) | 0.62 (0.07-1.29) | 17,434 (1,952-35,795) | 15.99 (1.8-33.06) | 2,202 (272-4,463) | 0.97 (0.12-1.98) | 60,389 (7,659-121,928) | 23.18 (2.89-46.74) | 1.46 (1.36 to 1.57) | 1.2 (1.1 to 1.3) | |
| **Iran (Islamic Republic of)** | 115 (14-231) | 0.45 (0.05-0.89) | 3,378 (414-6,699) | 11.52 (1.4-22.87) | 308 (41-609) | 0.4 (0.05-0.8) | 8,474 (1,128-16,599) | 10.03 (1.34-19.71) | -0.19 (-0.39 to 0.02) | -0.19 (-0.39 to 0.02) | |
| **Iraq** | 103 (12-193) | 1.35 (0.16-2.52) | 2,756 (321-5,189) | 34 (3.96-63.8) | 315 (34-639) | 1.44 (0.15-2.94) | 8,276 (875-16,829) | 33.16 (3.53-67.26) | 0.01 (-0.15 to 0.18) | -0.27 (-0.39 to -0.16) | |
| **Ireland** | 87 (10-183) | 2.13 (0.25-4.47) | 2,129 (244-4,458) | 54.28 (6.19-113.16) | 51 (6-112) | 0.65 (0.07-1.44) | 1,217 (130-2,702) | 16.33 (1.74-36.5) | -3.82 (-4.03 to -3.61) | -3.87 (-4.08 to -3.67) | |
| **Israel** | 53 (7-109) | 1.14 (0.14-2.34) | 1,419 (176-2,897) | 31.3 (3.86-63.68) | 65 (7-150) | 0.55 (0.06-1.26) | 1,607 (185-3,676) | 14.35 (1.65-32.82) | -2.22 (-2.55 to -1.88) | -2.35 (-2.7 to -2) | |
| **Italy** | 1,827 (210-3,455) | 2.11 (0.24-4) | 49,426 (5,708-93,090) | 59.56 (6.9-112.81) | 1,182 (147-2,356) | 0.86 (0.11-1.69) | 26,431 (3,308-51,899) | 21.82 (2.72-42.82) | -2.94 (-3.01 to -2.87) | -3.28 (-3.35 to -3.21) | |
| **Jamaica** | 11 (1-23) | 0.63 (0.06-1.31) | 274 (28-569) | 16.21 (1.65-33.64) | 13 (1-30) | 0.43 (0.05-0.96) | 327 (35-736) | 10.57 (1.12-23.81) | -1.75 (-2.15 to -1.35) | -1.95 (-2.37 to -1.53) | |
| **Japan** | 2,119 (281-4,121) | 1.25 (0.17-2.43) | 50,893 (6,762-97,124) | 29.67 (3.95-56.61) | 2,573 (324-5,225) | 0.65 (0.08-1.3) | 44,789 (5,761-89,750) | 14.08 (1.85-28.37) | -2.34 (-2.55 to -2.13) | -2.59 (-2.82 to -2.36) | |
| **Jordan** | 15 (2-28) | 1.13 (0.14-2.12) | 442 (57-829) | 29.23 (3.73-55.16) | 50 (6-107) | 0.67 (0.08-1.44) | 1,440 (164-3,075) | 16.87 (1.92-35.9) | -1.64 (-1.92 to -1.37) | -1.82 (-2.1 to -1.53) | |
| **Kazakhstan** | 268 (30-588) | 2.03 (0.23-4.47) | 8,111 (915-17,783) | 58.81 (6.54-128.88) | 122 (13-265) | 0.65 (0.07-1.41) | 3,464 (376-7,507) | 17.55 (1.93-38.09) | -3.52 (-3.72 to -3.32) | -3.82 (-4.01 to -3.63) | |
| **Kenya** | 3 (0-7) | 0.04 (0-0.09) | 97 (11-208) | 1.09 (0.13-2.32) | 10 (1-20) | 0.04 (0.01-0.09) | 298 (35-589) | 1.13 (0.13-2.25) | -0.1 (-0.35 to 0.15) | -0.07 (-0.33 to 0.18) | |
| **Kiribati** | 0 (0-1) | 0.73 (0.09-1.62) | 7 (1-16) | 17.91 (2.1-40.36) | 1 (0-2) | 1.09 (0.14-2.58) | 21 (3-50) | 26.58 (3.49-63.61) | 1.17 (0.83 to 1.52) | 1.15 (0.8 to 1.5) | |
| **Kuwait** | 6 (1-11) | 1.03 (0.13-1.96) | 168 (20-315) | 25.2 (3.11-47.73) | 14 (2-28) | 0.54 (0.07-1.03) | 401 (53-770) | 12.05 (1.61-23.13) | -1.68 (-2.01 to -1.36) | -2.04 (-2.35 to -1.73) | |
| **Kyrgyzstan** | 46 (5-92) | 1.5 (0.18-3) | 1,384 (159-2,725) | 43.58 (5.06-85.76) | 36 (5-73) | 0.75 (0.09-1.48) | 1,053 (133-2,094) | 19.6 (2.46-38.94) | -1.49 (-1.91 to -1.07) | -1.9 (-2.28 to -1.51) | |
| **Lao People's Democratic Republic** | 24 (3-61) | 1.19 (0.13-3) | 671 (76-1,700) | 30.17 (3.4-76.25) | 37 (4-90) | 0.86 (0.09-2.08) | 1,006 (105-2,462) | 20.36 (2.14-49.68) | -1.25 (-1.33 to -1.17) | -1.46 (-1.54 to -1.38) | |
| **Latvia** | 67 (6-145) | 1.85 (0.17-4) | 1,885 (178-4,110) | 52.56 (4.99-114.11) | 29 (3-67) | 0.76 (0.08-1.77) | 690 (73-1,593) | 19.82 (2.06-46.09) | -2.87 (-3.09 to -2.66) | -3.26 (-3.48 to -3.04) | |
| **Lebanon** | 38 (5-76) | 1.77 (0.25-3.58) | 1,007 (142-2,006) | 43.67 (6.13-87.33) | 106 (11-211) | 1.75 (0.17-3.46) | 2,375 (235-4,678) | 40.58 (4-80.22) | 0.64 (0.26 to 1.02) | 0.42 (0.06 to 0.78) | |
| **Lesotho** | 4 (0-10) | 0.48 (0.04-1.16) | 115 (10-282) | 12.87 (1.13-31.41) | 13 (1-29) | 1.13 (0.09-2.58) | 382 (31-863) | 31.83 (2.62-72.21) | 3.64 (3.22 to 4.07) | 3.76 (3.28 to 4.25) | |
| **Liberia** | 1 (0-2) | 0.08 (0.01-0.16) | 24 (3-50) | 2.01 (0.21-4.19) | 1 (0-3) | 0.06 (0.01-0.13) | 39 (4-83) | 1.59 (0.16-3.36) | -0.57 (-0.79 to -0.35) | -0.62 (-0.85 to -0.38) | |
| **Libya** | 35 (4-68) | 1.89 (0.25-3.73) | 928 (118-1,834) | 47.04 (6.05-93) | 101 (12-210) | 1.98 (0.24-4.11) | 2,838 (347-5,956) | 49.21 (6-103.03) | 0.41 (0.15 to 0.67) | 0.38 (0.13 to 0.64) | |
| **Lithuania** | 62 (6-136) | 1.35 (0.13-2.96) | 1,764 (167-3,880) | 38.84 (3.7-85.28) | 38 (4-85) | 0.69 (0.06-1.53) | 919 (85-2,045) | 18.2 (1.65-40.43) | -2.39 (-2.52 to -2.26) | -2.66 (-2.8 to -2.52) | |
| **Luxembourg** | 10 (1-21) | 1.91 (0.17-3.95) | 277 (25-577) | 53.8 (4.86-111.78) | 9 (1-19) | 0.85 (0.07-1.83) | 222 (19-479) | 22.1 (1.89-47.68) | -2.54 (-2.66 to -2.41) | -2.8 (-2.95 to -2.66) | |
| **Madagascar** | 8 (1-16) | 0.16 (0.02-0.31) | 237 (25-461) | 4.26 (0.45-8.28) | 10 (1-20) | 0.08 (0.01-0.17) | 313 (34-652) | 2.26 (0.25-4.64) | -2.27 (-2.49 to -2.04) | -2.2 (-2.43 to -1.98) | |
| **Malawi** | 1 (0-3) | 0.03 (0-0.07) | 42 (5-89) | 0.97 (0.12-2.04) | 3 (0-8) | 0.04 (0-0.1) | 105 (11-249) | 1.2 (0.13-2.84) | 0.4 (0.1 to 0.71) | 0.46 (0.14 to 0.78) | |
| **Malaysia** | 77 (9-177) | 0.86 (0.1-1.97) | 2106 (251-4843) | 21.65 (2.58-49.95) | 213 (20-525) | 0.76 (0.07-1.89) | 5,610 (521-14,199) | 18.81 (1.76-47.32) | -0.45 (-0.87 to -0.03) | -0.5 (-0.83 to -0.16) | |
| **Maldives** | 1 (0-2) | 0.98 (0.1-2.15) | 23 (2-49) | 23.76 (2.5-51.2) | 1 (0-3) | 0.4 (0.04-0.91) | 31 (3-72) | 8.66 (0.85-19.9) | -3.55 (-3.75 to -3.34) | -3.95 (-4.15 to -3.74) | |
| **Mali** | 3 (0-7) | 0.09 (0.01-0.18) | 97 (13-200) | 2.23 (0.3-4.58) | 11 (1-24) | 0.12 (0.01-0.28) | 300 (30-685) | 3.12 (0.32-7.02) | 1.44 (1.36 to 1.51) | 1.32 (1.25 to 1.38) | |
| **Malta** | 5 (1-11) | 1.27 (0.16-2.5) | 147 (18-288) | 34.43 (4.28-67.55) | 6 (1-12) | 0.63 (0.07-1.33) | 137 (15-287) | 17.45 (2-35.87) | -2.18 (-2.31 to -2.05) | -2.11 (-2.27 to -1.96) | |
| **Marshall Islands** | 0 (0-0) | 1.12 (0.1-2.72) | 5 (0-11) | 27.3 (2.52-66.39) | 0 (0-1) | 1.23 (0.11-3.2) | 11 (1-29) | 29.44 (2.59-75.73) | 0.29 (0.21 to 0.37) | 0.23 (0.15 to 0.31) | |
| **Mauritania** | 3 (0-7) | 0.28 (0.04-0.68) | 73 (9-179) | 7.06 (0.9-17) | 5 (1-10) | 0.22 (0.03-0.49) | 116 (16-254) | 5.16 (0.7-11.45) | -0.97 (-1.16 to -0.78) | -1.17 (-1.37 to -0.97) | |
| **Mauritius** | 5 (1-10) | 0.65 (0.07-1.5) | 119 (13-274) | 15.67 (1.74-35.87) | 9 (1-21) | 0.5 (0.05-1.13) | 222 (22-505) | 11.82 (1.19-26.82) | -1.03 (-1.29 to -0.76) | -1.08 (-1.36 to -0.8) | |
| **Mexico** | 174 (22-326) | 0.44 (0.06-0.82) | 4,417 (572-8,306) | 10.04 (1.3-18.87) | 152 (20-291) | 0.12 (0.02-0.24) | 3,772 (481-7,263) | 2.9 (0.37-5.57) | -4.63 (-4.83 to -4.42) | -4.55 (-4.76 to -4.33) | |
| **Micronesia (Federated States of)** | 1 (0-1) | 1.17 (0.13-2.44) | 15 (2-30) | 28.92 (3.43-60.28) | 1 (0-2) | 1.31 (0.15-2.74) | 26 (3-54) | 32.12 (3.75-67.27) | 0.37 (0.34 to 0.4) | 0.37 (0.34 to 0.41) | |
| **Monaco** | 1 (0-3) | 2.22 (0.23-4.84) | 34 (3-74) | 63.27 (6.59-137.09) | 2 (0-4) | 2.01 (0.2-4.41) | 42 (4-94) | 56.25 (5.72-125.19) | -0.21 (-0.53 to 0.1) | -0.29 (-0.59 to 0.01) | |
| **Mongolia** | 11 (1-27) | 1.08 (0.14-2.61) | 310 (40-740) | 28.14 (3.7-67.42) | 21 (2-50) | 0.93 (0.11-2.19) | 608 (70-1,437) | 23.34 (2.71-55.51) | -1 (-1.2 to -0.8) | -1.11 (-1.32 to -0.9) | |
| **Montenegro** | 20 (3-39) | 3.19 (0.4-6.17) | 563 (73-1,093) | 85.63 (10.92-167.15) | 34 (4-70) | 3.45 (0.38-7.06) | 828 (91-1,683) | 84.34 (9.36-170.96) | 0.39 (0.11 to 0.66) | 0.09 (-0.25 to 0.43) | |
| **Morocco** | 114 (13-223) | 0.82 (0.1-1.6) | 2,966 (342-5,791) | 20.3 (2.34-39.58) | 262 (31-565) | 0.77 (0.09-1.66) | 6,624 (790-14,148) | 18.33 (2.17-39.29) | -0.34 (-0.53 to -0.15) | -0.45 (-0.64 to -0.26) | |
| **Mozambique** | 5 (1-9) | 0.08 (0.01-0.16) | 124 (15-245) | 1.96 (0.24-3.86) | 10 (1-20) | 0.09 (0.01-0.19) | 277 (31-575) | 2.27 (0.25-4.71) | 0.9 (0.74 to 1.05) | 1.01 (0.84 to 1.19) | |
| **Myanmar** | 222 (24-510) | 0.98 (0.11-2.25) | 6,254 (690-14,410) | 25.08 (2.76-58) | 243 (29-477) | 0.51 (0.06-1.02) | 6,401 (758-12,680) | 12.45 (1.47-24.59) | -2.11 (-2.25 to -1.98) | -2.3 (-2.44 to -2.17) | |
| **Namibia** | 1 (0-2) | 0.15 (0.02-0.3) | 30 (4-59) | 4.14 (0.54-8.24) | 2 (0-4) | 0.14 (0.02-0.27) | 57 (7-118) | 3.66 (0.46-7.48) | -0.62 (-0.92 to -0.33) | -0.7 (-1.03 to -0.37) | |
| **Nauru** | 0 (0-0) | 2.06 (0.26-4.57) | 2 (0-5) | 49.91 (6.25-109.36) | 0 (0-0) | 1.8 (0.21-3.67) | 3 (0-6) | 44.75 (5.09-89.98) | -0.54 (-0.62 to -0.45) | -0.48 (-0.59 to -0.37) | |
| **Nepal** | 16 (2-34) | 0.17 (0.02-0.37) | 459 (48-959) | 4.46 (0.47-9.39) | 34 (4-70) | 0.15 (0.02-0.31) | 908 (116-1,886) | 3.71 (0.47-7.67) | -0.51 (-0.73 to -0.29) | -0.62 (-0.85 to -0.38) | |
| **Netherlands** | 315 (34-680) | 1.65 (0.18-3.54) | 8,527 (916-18,265) | 46.26 (4.95-98.69) | 242 (28-535) | 0.72 (0.08-1.59) | 5,911 (687-12,988) | 19.14 (2.21-41.98) | -2.14 (-2.36 to -1.92) | -2.33 (-2.56 to -2.09) | |
| **New Zealand** | 44 (5-101) | 1.15 (0.13-2.63) | 1,200 (140-2,744) | 32.25 (3.8-73.89) | 37 (4-87) | 0.45 (0.05-1.07) | 944 (109-2,220) | 12.36 (1.44-29.09) | -2.91 (-3.04 to -2.79) | -2.99 (-3.13 to -2.85) | |
| **Nicaragua** | 3 (0-6) | 0.18 (0.02-0.4) | 74 (8-162) | 4.53 (0.49-9.89) | 7 (1-18) | 0.15 (0.01-0.37) | 193 (19-461) | 3.78 (0.37-8.97) | -0.29 (-0.45 to -0.14) | -0.34 (-0.49 to -0.2) | |
| **Niger** | 2 (0-4) | 0.07 (0.01-0.16) | 56 (6-124) | 1.83 (0.19-4.02) | 4 (0-10) | 0.06 (0.01-0.12) | 125 (14-272) | 1.38 (0.15-3.01) | -0.6 (-0.81 to -0.4) | -0.76 (-0.98 to -0.54) | |
| **Nigeria** | 7 (1-14) | 0.02 (0-0.03) | 194 (22-385) | 0.41 (0.05-0.81) | 17 (2-34) | 0.02 (0-0.04) | 493 (54-994) | 0.48 (0.05-0.96) | 0.84 (0.69 to 0.99) | 0.7 (0.56 to 0.84) | |
| **Niue** | 0 (0-0) | 1.05 (0.11-2.51) | 1 (0-1) | 25.04 (2.6-60.44) | 0 (0-0) | 1.24 (0.14-3.24) | 1 (0-2) | 28.64 (3.26-73.3) | 0.45 (0.36 to 0.55) | 0.32 (0.23 to 0.4) | |
| **North Macedonia** | 52 (7-103) | 2.74 (0.35-5.38) | 1,438 (180-2,882) | 71.95 (9.03-143.31) | 86 (10-179) | 2.55 (0.29-5.31) | 2,184 (241-4,578) | 63.26 (7.03-132.65) | -0.25 (-0.63 to 0.13) | -0.43 (-0.8 to -0.07) | |
| **Northern Mariana Islands** | 0 (0-1) | 1.93 (0.23-4.41) | 8 (1-19) | 44.22 (5.13-98.68) | 1 (0-2) | 1.51 (0.17-3.54) | 19 (2-45) | 34.17 (3.93-80.84) | -0.96 (-1.07 to -0.84) | -0.97 (-1.08 to -0.86) | |
| **Norway** | 58 (7-110) | 0.94 (0.12-1.78) | 1,467 (192-2,795) | 25.88 (3.45-49.06) | 50 (6-101) | 0.51 (0.07-1.03) | 1,145 (148-2,303) | 12.66 (1.64-25.27) | -2.13 (-2.43 to -1.84) | -2.48 (-2.8 to -2.16) | |
| **Oman** | 2 (0-4) | 0.27 (0.03-0.65) | 51 (6-120) | 6.97 (0.81-16.5) | 4 (0-8) | 0.2 (0.02-0.47) | 103 (10-244) | 4.58 (0.47-10.88) | -0.8 (-1.16 to -0.45) | -1.13 (-1.52 to -0.73) | |
| **Pakistan** | 397 (45-814) | 0.72 (0.08-1.48) | 10,785 (1,254-22,133) | 18.31 (2.12-37.51) | 744 (81-1,612) | 0.62 (0.07-1.34) | 21,621 (2,358-46,423) | 15.88 (1.73-34.37) | -0.78 (-1.15 to -0.4) | -0.73 (-1.12 to -0.35) | |
| **Palau** | 0 (0-0) | 1.77 (0.2-4.54) | 4 (0-10) | 40.98 (4.6-104.15) | 0 (0-1) | 1.72 (0.17-4.27) | 9 (1-23) | 38.84 (3.75-96.93) | -0.11 (-0.18 to -0.04) | -0.23 (-0.3 to -0.16) | |
| **Palestine** | 12 (1-23) | 1.4 (0.18-2.72) | 304 (38-596) | 33.81 (4.27-65.7) | 27 (3-56) | 1.1 (0.12-2.27) | 768 (88-1,604) | 27.02 (3.06-56.01) | -0.96 (-1.15 to -0.77) | -0.9 (-1.08 to -0.72) | |
| **Panama** | 6 (1-14) | 0.4 (0.04-0.93) | 153 (14-353) | 10.03 (0.92-23.07) | 7 (1-17) | 0.15 (0.02-0.38) | 161 (16-399) | 3.65 (0.37-9.03) | -3.39 (-3.55 to -3.23) | -3.52 (-3.7 to -3.33) | |
| **Papua New Guinea** | 10 (1-22) | 0.61 (0.06-1.35) | 276 (28-619) | 14.49 (1.47-32.27) | 34 (4-80) | 0.76 (0.09-1.81) | 953 (117-2,200) | 17.89 (2.18-41.6) | 0.68 (0.62 to 0.73) | 0.6 (0.53 to 0.66) | |
| **Paraguay** | 9 (1-22) | 0.42 (0.04-1.03) | 244 (25-575) | 10.63 (1.09-25.09) | 27 (3-68) | 0.47 (0.05-1.19) | 676 (69-1,739) | 11.29 (1.15-28.89) | 0.43 (0.29 to 0.57) | 0.2 (0.06 to 0.35) | |
| **Peru** | 29 (3-54) | 0.25 (0.03-0.46) | 815 (88-1,506) | 6.4 (0.69-11.85) | 34 (4-70) | 0.1 (0.01-0.21) | 894 (97-1811) | 2.59 (0.28-5.25) | -3.89 (-4.34 to -3.43) | -3.93 (-4.39 to -3.48) | |
| **Philippines** | 309 (41-578) | 1.15 (0.15-2.17) | 8,538 (1,113-15,994) | 27.13 (3.55-50.54) | 579 (70-1105) | 0.72 (0.09-1.37) | 15,890 (1,924-30,412) | 17.94 (2.17-34.26) | -1.58 (-1.72 to -1.43) | -1.46 (-1.62 to -1.3) | |
| **Poland** | 1,319 (175-2,499) | 2.99 (0.39-5.69) | 37,664 (4,970-71,729) | 86.07 (11.25-164.28) | 1,082 (161-2,271) | 1.5 (0.22-3.14) | 25,650 (3,760-53,477) | 37.46 (5.53-77.4) | -2.48 (-2.67 to -2.28) | -2.91 (-3.14 to -2.69) | |
| **Portugal** | 132 (12-274) | 0.97 (0.09-2.02) | 3,616 (326-7,476) | 27.58 (2.49-56.49) | 128 (11-283) | 0.58 (0.05-1.29) | 3,169 (261-6998) | 16.18 (1.34-36.02) | -1.74 (-1.96 to -1.52) | -1.81 (-2.1 to -1.52) | |
| **Puerto Rico** | 12 (1-28) | 0.34 (0.04-0.77) | 297 (32-674) | 8.27 (0.89-18.72) | 12 (1-26) | 0.16 (0.01-0.37) | 251 (23-565) | 4.1 (0.37-9.21) | -2.3 (-2.42 to -2.17) | -2.29 (-2.41 to -2.16) | |
| **Qatar** | 2 (0-3) | 1.68 (0.22-3.37) | 47 (6-97) | 37.95 (5.11-76.65) | 7 (1-13) | 0.86 (0.11-1.74) | 208 (26-418) | 18.33 (2.22-37.15) | -2.23 (-2.81 to -1.64) | -2.35 (-2.88 to -1.82) | |
| **Republic of Korea** | 493 (59-973) | 1.67 (0.2-3.29) | 14,331 (1,701-28,579) | 43.01 (5.16-85.41) | 834 (91-1,966) | 0.88 (0.1-2.07) | 17,099 (1,893-39,430) | 18.1 (1.99-41.72) | -2.75 (-3.16 to -2.34) | -3.33 (-3.66 to -3) | |
| **Republic of Moldova** | 63 (8-122) | 1.36 (0.16-2.64) | 1,932 (235-3,787) | 41.14 (5-80.14) | 44 (5-88) | 0.74 (0.09-1.47) | 1,249 (148-2,496) | 21.63 (2.57-43.05) | -1.46 (-1.75 to -1.17) | -1.63 (-1.88 to -1.37) | |
| **Romania** | 398 (43-825) | 1.38 (0.15-2.84) | 12,677 (1,390-26,136) | 44 (4.83-90.35) | 443 (34-1,010) | 1.29 (0.1-2.95) | 11,884 (921-26,998) | 36.87 (2.9-84.46) | -0.42 (-0.65 to -0.19) | -0.81 (-1.04 to -0.58) | |
| **Russian Federation** | 2,421 (289-4,630) | 1.29 (0.15-2.46) | 71,807 (8,469-137,367) | 38.21 (4.52-72.88) | 2,040 (237-4,063) | 0.85 (0.1-1.68) | 54,191 (6,289-107,980) | 23.12 (2.65-46.14) | -1.41 (-1.66 to -1.15) | -1.69 (-1.94 to -1.44) | |
| **Rwanda** | 5 (1-13) | 0.16 (0.02-0.43) | 151 (17-409) | 4.65 (0.53-12.53) | 8 (1-24) | 0.13 (0.01-0.36) | 254 (26-715) | 3.49 (0.37-9.76) | -1.36 (-1.74 to -0.98) | -1.68 (-2.08 to -1.28) | |
| **Saint Kitts and Nevis** | 0 (0-0) | 0.3 (0.04-0.66) | 3 (0-6) | 7.7 (0.98-16.8) | 0 (0-0) | 0.17 (0.02-0.41) | 3 (0-8) | 4.01 (0.47-9.89) | -1.81 (-1.94 to -1.68) | -2.02 (-2.16 to -1.88) | |
| **Saint Lucia** | 0 (0-1) | 0.36 (0.04-0.84) | 8 (1-18) | 9.13 (1.01-21.21) | 0 (0-1) | 0.14 (0.01-0.35) | 9 (1-22) | 3.61 (0.37-8.89) | -3.57 (-3.87 to -3.27) | -3.45 (-3.71 to -3.2) | |
| **Saint Vincent and the Grenadines** | 0 (0-0) | 0.19 (0.02-0.45) | 3 (0-8) | 4.84 (0.52-11.18) | 0 (0-1) | 0.16 (0.02-0.39) | 6 (1-14) | 4.12 (0.39-9.74) | -0.43 (-0.57 to -0.29) | -0.38 (-0.52 to -0.25) | |
| **Samoa** | 0 (0-1) | 0.6 (0.06-1.32) | 13 (1-29) | 14.53 (1.61-32.63) | 1 (0-2) | 0.64 (0.06-1.35) | 24 (2-50) | 15.66 (1.55-33.05) | 0.25 (0.21 to 0.28) | 0.29 (0.24 to 0.34) | |
| **San Marino** | 1 (0-1) | 1.52 (0.16-3.08) | 13 (1-26) | 40.74 (4.4-82.29) | 0 (0-1) | 0.56 (0.07-1.26) | 9 (1-21) | 15.13 (1.77-34.59) | -2.33 (-2.64 to -2.01) | -2.31 (-2.62 to -2) | |
| **Sao Tome and Principe** | 0 (0-0) | 0.13 (0.01-0.32) | 2 (0-6) | 3.48 (0.38-8.81) | 0 (0-0) | 0.12 (0.01-0.27) | 4 (0-9) | 3.11 (0.33-7.15) | -0.65 (-0.85 to -0.45) | -0.77 (-0.98 to -0.55) | |
| **Saudi Arabia** | 23 (3-45) | 0.41 (0.05-0.81) | 642 (82-1,292) | 10.11 (1.27-20.39) | 73 (9-150) | 0.39 (0.05-0.8) | 2,309 (299-4,678) | 9.55 (1.22-19.76) | -0.22 (-0.42 to -0.02) | -0.23 (-0.42 to -0.03) | |
| **Senegal** | 11 (1-24) | 0.35 (0.04-0.79) | 285 (34-647) | 8.5 (1-19.08) | 22 (2-53) | 0.31 (0.03-0.72) | 581 (56-1,378) | 7.19 (0.69-17.02) | -0.43 (-0.61 to -0.25) | -0.53 (-0.71 to -0.34) | |
| **Serbia** | 310 (41-637) | 2.68 (0.36-5.53) | 8,917 (1,159-18,275) | 72.93 (9.61-149.76) | 376 (41-842) | 2.32 (0.26-5.22) | 9,407 (1,030-21,049) | 61.52 (6.84-137.02) | -0.3 (-0.67 to 0.07) | -0.45 (-0.85 to -0.04) | |
| **Seychelles** | 1 (0-1) | 0.98 (0.11-2.12) | 14 (2-29) | 24.12 (2.76-52.21) | 1 (0-2) | 0.64 (0.07-1.47) | 18 (2-41) | 15.02 (1.58-33.68) | -1.53 (-1.71 to -1.36) | -1.71 (-1.89 to -1.53) | |
| **Sierra Leone** | 3 (0-7) | 0.17 (0.02-0.33) | 85 (10-169) | 4.07 (0.49-8.05) | 5 (1-10) | 0.14 (0.01-0.28) | 128 (15-264) | 3.22 (0.36-6.65) | -0.43 (-0.58 to -0.29) | -0.47 (-0.61 to -0.32) | |
| **Singapore** | 25 (2-52) | 1.15 (0.11-2.41) | 683 (68-1,434) | 28.78 (2.85-60.61) | 31 (3-69) | 0.36 (0.04-0.81) | 709 (80-1,562) | 8.13 (0.92-18.08) | -3.74 (-3.93 to -3.55) | -4.07 (-4.25 to -3.89) | |
| **Slovakia** | 146 (16-310) | 2.45 (0.27-5.19) | 4,170 (464-8,838) | 70.97 (7.9-150.03) | 119 (11-262) | 1.26 (0.12-2.76) | 3,063 (294-6,671) | 33.58 (3.24-73.12) | -1.97 (-2.14 to -1.8) | -2.25 (-2.42 to -2.08) | |
| **Slovenia** | 63 (8-117) | 2.53 (0.33-4.69) | 1,824 (242-3,383) | 73.58 (9.77-135.96) | 66 (7-131) | 1.5 (0.15-3) | 1,497 (155-2,978) | 37.15 (3.85-73.51) | -1.49 (-1.69 to -1.28) | -1.98 (-2.22 to -1.75) | |
| **Solomon Islands** | 1 (0-3) | 0.86 (0.09-2.13) | 34 (4-84) | 22.09 (2.43-54.33) | 3 (0-8) | 0.98 (0.11-2.27) | 103 (11-240) | 25.83 (2.83-59.28) | 0.55 (0.27 to 0.83) | 0.66 (0.37 to 0.95) | |
| **Somalia** | 3 (0-7) | 0.11 (0.01-0.28) | 87 (11-218) | 3.02 (0.37-7.63) | 6 (1-15) | 0.09 (0.01-0.24) | 181 (20-486) | 2.44 (0.28-6.49) | -0.52 (-0.59 to -0.45) | -0.59 (-0.66 to -0.52) | |
| **South Africa** | 198 (23-384) | 0.94 (0.11-1.82) | 6,007 (711-11,794) | 26.52 (3.13-51.81) | 257 (33-495) | 0.54 (0.07-1.05) | 7,427 (953-14,258) | 14.71 (1.88-28.25) | -1.87 (-2.06 to -1.68) | -1.99 (-2.18 to -1.81) | |
| **South Sudan** | 3 (0-8) | 0.13 (0.02-0.3) | 96 (11-219) | 3.54 (0.41-8.07) | 5 (1-11) | 0.13 (0.01-0.28) | 150 (16-322) | 3.34 (0.37-7.19) | -0.08 (-0.18 to 0.02) | -0.19 (-0.33 to -0.04) | |
| **Spain** | 1,053 (126-2,095) | 1.98 (0.24-3.94) | 27,779 (3,300-55,085) | 54.62 (6.55-108.34) | 857 (95-1,893) | 0.94 (0.1-2.1) | 20,665 (23,07-46,253) | 24.79 (2.78-55.79) | -2.46 (-2.7 to -2.21) | -2.64 (-2.92 to -2.37) | |
| **Sri Lanka** | 26 (3-56) | 0.25 (0.03-0.54) | 727 (77-1,558) | 6.25 (0.66-13.49) | 46 (5-93) | 0.17 (0.02-0.34) | 1,185 (135-2,389) | 4.25 (0.48-8.55) | -1.22 (-1.34 to -1.09) | -1.18 (-1.32 to -1.04) | |
| **Sudan** | 43 (5-95) | 0.47 (0.05-1.05) | 1,205 (136-2,644) | 12.09 (1.36-26.41) | 94 (10-199) | 0.49 (0.05-1.03) | 2,716 (276-5,823) | 12.3 (1.27-26.1) | 0.21 (0.16 to 0.26) | 0.13 (0.07 to 0.18) | |
| **Suriname** | 2 (0-4) | 0.68 (0.07-1.53) | 46 (5-106) | 17.2 (1.91-39.14) | 3 (0-7) | 0.39 (0.04-1.09) | 67 (6-188) | 10.12 (0.98-28.3) | -1.9 (-2.26 to -1.53) | -1.9 (-2.25 to -1.55) | |
| **Sweden** | 102 (13-210) | 0.75 (0.1-1.55) | 2,590 (330-5,340) | 20.83 (2.67-43) | 77 (9-159) | 0.36 (0.04-0.75) | 1,608 (191-3303) | 8.56 (1.03-17.59) | -2.19 (-2.4 to -1.98) | -2.66 (-2.91 to -2.42) | |
| **Switzerland** | 123 (12-250) | 1.28 (0.13-2.59) | 3,342 (328-6,719) | 36.52 (3.56-73.18) | 79 (8-177) | 0.46 (0.04-1.03) | 1,889 (184-4,219) | 12.1 (1.18-26.92) | -3.28 (-3.36 to -3.2) | -3.6 (-3.69 to -3.51) | |
| **Syrian Arab Republic** | 49 (5-96) | 0.93 (0.09-1.87) | 1,442 (146-2,859) | 24.6 (2.48-48.56) | 90 (9-198) | 0.69 (0.06-1.51) | 2,529 (244-5,546) | 17.29 (1.65-38.03) | -1.26 (-1.42 to -1.11) | -1.42 (-1.57 to -1.26) | |
| **Taiwan (Province of China)** | 295 (37-563) | 1.89 (0.24-3.61) | 7,957 (988-15,225) | 46.88 (5.84-89.5) | 656 (80-1,384) | 1.54 (0.19-3.23) | 14,470 (1,762-30,510) | 34.87 (4.24-73.07) | -0.83 (-1.15 to -0.5) | -1.01 (-1.27 to -0.75) | |
| **Tajikistan** | 34 (4-63) | 1.2 (0.15-2.23) | 1,008 (127-1,890) | 33.9 (4.3-63.4) | 24 (3-55) | 0.39 (0.05-0.91) | 756 (95-1,732) | 10.77 (1.33-24.59) | -3.42 (-3.68 to -3.15) | -3.6 (-3.84 to -3.35) | |
| **Thailand** | 450 (56-1,048) | 1.33 (0.17-3.09) | 12,371 (1,527-29,007) | 32.47 (4.04-75.88) | 798 (96-1,855) | 0.74 (0.09-1.71) | 18,709 (2,257-43,241) | 17.5 (2.12-40.18) | -2.57 (-2.77 to -2.37) | -2.7 (-2.92 to -2.48) | |
| **Timor-Leste** | 1 (0-3) | 0.47 (0.06-1.09) | 34 (4-78) | 11.2 (1.49-26.32) | 4 (0-9) | 0.5 (0.05-1.12) | 103 (11-234) | 11.7 (1.22-26.58) | 0.16 (-0.01 to 0.33) | 0.06 (-0.13 to 0.26) | |
| **Togo** | 3 (0-7) | 0.24 (0.03-0.55) | 92 (10-206) | 6.57 (0.72-14.77) | 10 (1-22) | 0.24 (0.02-0.54) | 306 (30-687) | 6.59 (0.66-14.93) | 0.04 (-0.03 to 0.11) | 0.16 (0.08 to 0.24) | |
| **Tokelau** | 0 (0-0) | 0.94 (0.1-2.25) | 0 (0-1) | 21.68 (2.15-52.1) | 0 (0-0) | 0.94 (0.09-2.54) | 0 (0-1) | 21.85 (1.92-59.17) | -0.03 (-0.09 to 0.03) | -0.05 (-0.1 to 0) | |
| **Tonga** | 1 (0-2) | 1.36 (0.13-3.35) | 18 (2-44) | 31.35 (3.05-77.9) | 1 (0-3) | 1.49 (0.14-3.83) | 28 (3-71) | 34.16 (3.19-88.37) | 0.22 (0.02 to 0.43) | 0.22 (0.03 to 0.41) | |
| **Trinidad and Tobago** | 3 (0-7) | 0.4 (0.04-0.9) | 86 (9-192) | 10.06 (1.07-22.47) | 5 (0-11) | 0.24 (0.02-0.56) | 124 (12-286) | 6.31 (0.61-14.56) | -2.03 (-2.24 to -1.81) | -1.96 (-2.17 to -1.74) | |
| **Tunisia** | 72 (8-142) | 1.45 (0.17-2.84) | 1,881 (226-3,681) | 35.43 (4.2-69.43) | 143 (14-312) | 1.05 (0.1-2.31) | 3,765 (363-8,070) | 26.81 (2.59-57.4) | -1.52 (-1.72 to -1.31) | -1.34 (-1.54 to -1.15) | |
| **Turkey** | 1,268 (131-2,448) | 3.7 (0.39-7.14) | 35,250 (3,617-67,982) | 94.58 (9.81-182.63) | 2,045 (213-4,246) | 2.18 (0.23-4.51) | 51,114 (5,475-106,541) | 52.53 (5.62-108.99) | -1.91 (-2.15 to -1.66) | -2.15 (-2.38 to -1.92) | |
| **Turkmenistan** | 25 (3-47) | 1.22 (0.15-2.34) | 751 (93-1,430) | 35.16 (4.37-67.3) | 25 (3-53) | 0.58 (0.06-1.23) | 759 (82-1,622) | 16.42 (1.78-35.16) | -2.32 (-2.7 to -1.93) | -2.41 (-2.8 to -2.02) | |
| **Tuvalu** | 0 (0-0) | 1.03 (0.1-2.59) | 2 (0-5) | 25.38 (2.45-63.81) | 0 (0-0) | 1.12 (0.12-2.65) | 3 (0-7) | 26.8 (2.92-63.4) | 0.15 (0.07 to 0.23) | 0.08 (0.01 to 0.15) | |
| **Uganda** | 5 (1-10) | 0.07 (0.01-0.15) | 132 (15-267) | 1.91 (0.22-3.89) | 10 (1-22) | 0.07 (0.01-0.15) | 304 (39-642) | 1.83 (0.23-3.83) | -0.89 (-1.27 to -0.51) | -1.02 (-1.44 to -0.6) | |
| **Ukraine** | 1,507 (182-3,029) | 2.06 (0.25-4.13) | 42,233 (5,035-84,842) | 58.41 (6.96-116.95) | 535 (67-1155) | 0.7 (0.09-1.52) | 14,616 (1,876-31,588) | 20.09 (2.57-43.29) | -3.73 (-3.91 to -3.55) | -3.75 (-3.93 to -3.58) | |
| **United Arab Emirates** | 6 (1-12) | 1.37 (0.14-2.84) | 183 (20-376) | 33.91 (3.53-70.79) | 28 (4-57) | 0.94 (0.12-1.94) | 903 (118-1,867) | 19.63 (2.43-40.7) | -0.03 (-0.51 to 0.45) | -0.89 (-1.33 to -0.44) | |
| **United Kingdom** | 1,697 (206-3,281) | 2 (0.24-3.83) | 43602 (5,285-83,348) | 54.77 (6.65-104.03) | 594 (82-1,217) | 0.48 (0.07-0.99) | 14,246 (1,953-29,417) | 12.64 (1.73-25.89) | -4.62 (-4.68 to -4.55) | -4.79 (-4.86 to -4.73) | |
| **United Republic of Tanzania** | 14 (2-28) | 0.13 (0.01-0.26) | 399 (45-792) | 3.37 (0.39-6.7) | 25 (3-52) | 0.1 (0.01-0.21) | 721 (92-1,513) | 2.51 (0.32-5.3) | -1.26 (-1.36 to -1.15) | -1.26 (-1.36 to -1.15) | |
| **United States of America** | 6,175 (736-11,968) | 2.03 (0.24-3.92) | 164,857 (19,533-316,633) | 56.92 (6.7-109.1) | 3984 (523-7939) | 0.68 (0.09-1.36) | 93,718 (12,138-184,055) | 16.97 (2.19-33.37) | -3.75 (-3.91 to -3.59) | -4.11 (-4.26 to -3.96) | |
| **United States Virgin Islands** | 0 (0-1) | 0.56 (0.05-1.28) | 13 (1-30) | 14.14 (1.37-32.6) | 1 (0-2) | 0.39 (0.04-0.87) | 16 (1-36) | 9.72 (0.89-22.31) | -1.04 (-1.22 to -0.86) | -0.98 (-1.14 to -0.83) | |
| **Uruguay** | 76 (8-158) | 1.99 (0.21-4.12) | 2032 (217-4,195) | 54.91 (5.89-112.85) | 65 (8-134) | 1.25 (0.16-2.57) | 1,625 (209-3,348) | 33.44 (4.32-68.79) | -1.89 (-2.1 to -1.69) | -1.97 (-2.16 to -1.79) | |
| **Uzbekistan** | 73 (8-169) | 0.61 (0.07-1.43) | 2,261 (240-5,205) | 18.19 (1.94-41.87) | 83 (9-193) | 0.3 (0.03-0.7) | 2,527 (267-5,839) | 8.28 (0.87-19.09) | -2.2 (-2.44 to -1.96) | -2.49 (-2.74 to -2.24) | |
| **Vanuatu** | 0 (0-1) | 0.42 (0.05-0.97) | 7 (1-16) | 10.32 (1.3-24.17) | 1 (0-1) | 0.39 (0.05-0.82) | 18 (2-38) | 9.56 (1.1-20.22) | -0.4 (-0.47 to -0.32) | -0.39 (-0.47 to -0.31) | |
| **Venezuela (Bolivarian Republic of)** | 58 (6-115) | 0.61 (0.07-1.22) | 1,601 (176-3,171) | 15.57 (1.7-30.83) | 115 (13-235) | 0.39 (0.04-0.78) | 2,921 (320-6,017) | 9.42 (1.04-19.33) | -1.51 (-1.65 to -1.38) | -1.71 (-1.85 to -1.57) | |
| **Viet Nam** | 369 (40-914) | 0.92 (0.1-2.28) | 9,905 (1,077-24,563) | 23.75 (2.58-58.68) | 1,036 (111-2,368) | 1.03 (0.11-2.37) | 28,418 (3,000-64,523) | 26.35 (2.81-60.31) | -0.01 (-0.31 to 0.3) | 0.03 (-0.24 to 0.3) | |
| **Yemen** | 30 (4-69) | 0.61 (0.07-1.42) | 880 (107-2,095) | 16.12 (1.94-37.76) | 95 (9-219) | 0.71 (0.07-1.62) | 2,681 (268-6,155) | 17.33 (1.7-39.79) | 0.67 (0.54 to 0.8) | 0.42 (0.3 to 0.54) | |
| **Zambia** | 4 (0-9) | 0.15 (0.02-0.3) | 128 (14-257) | 4.03 (0.44-8.05) | 12 (1-25) | 0.17 (0.02-0.34) | 385 (42-777) | 4.6 (0.49-9.22) | 0.16 (0.04 to 0.29) | 0.23 (0.1 to 0.36) | |
| **Zimbabwe** | 15 (2-35) | 0.36 (0.04-0.88) | 417 (48-1,016) | 9.41 (1.09-22.78) | 27 (3-67) | 0.37 (0.03-0.93) | 836 (81-2,111) | 10.14 (0.96-25.34) | 0.1 (-0.39 to 0.6) | 0.34 (-0.19 to 0.88) | |


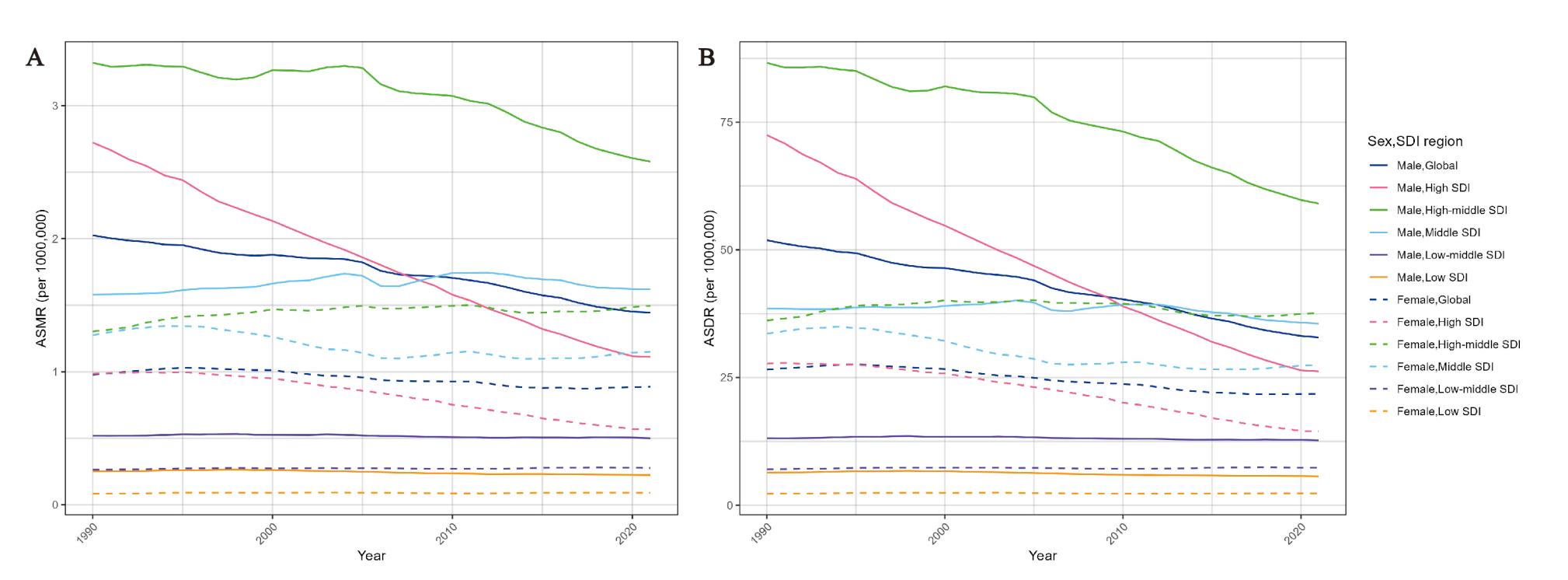


Figure S1. The global burden of ASMR (A), ASDR (B) attributable to second-hand smoke for male and female from 1990 to 2021 by SDI region.ASMR, age-standardized mortality rate; ASDR, age-standardized DALYs rate; DALYs, disability-adjusted life-year; SDI, sociodemographic index.


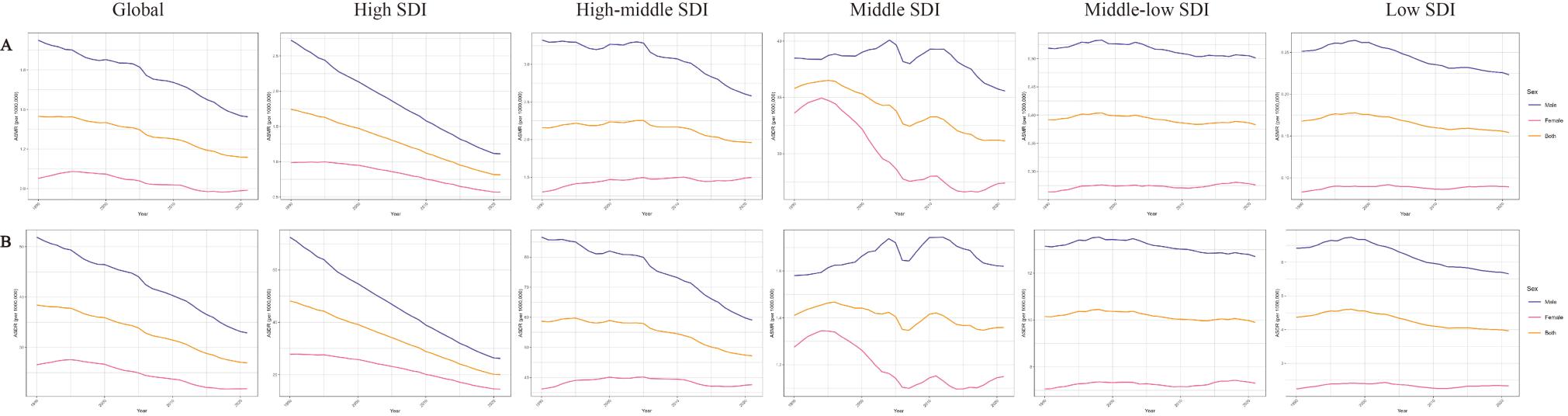


Figure S2. The global burden of ASMR (A), ASDR (B) attributable to second-hand smoke from 1990 to 2021 by sex and SDI region.ASMR, age-standardized mortality rate; ASDR, age-standardized DALYs rate; DALYs, disability-adjusted life-year; SDI, sociodemographic index.


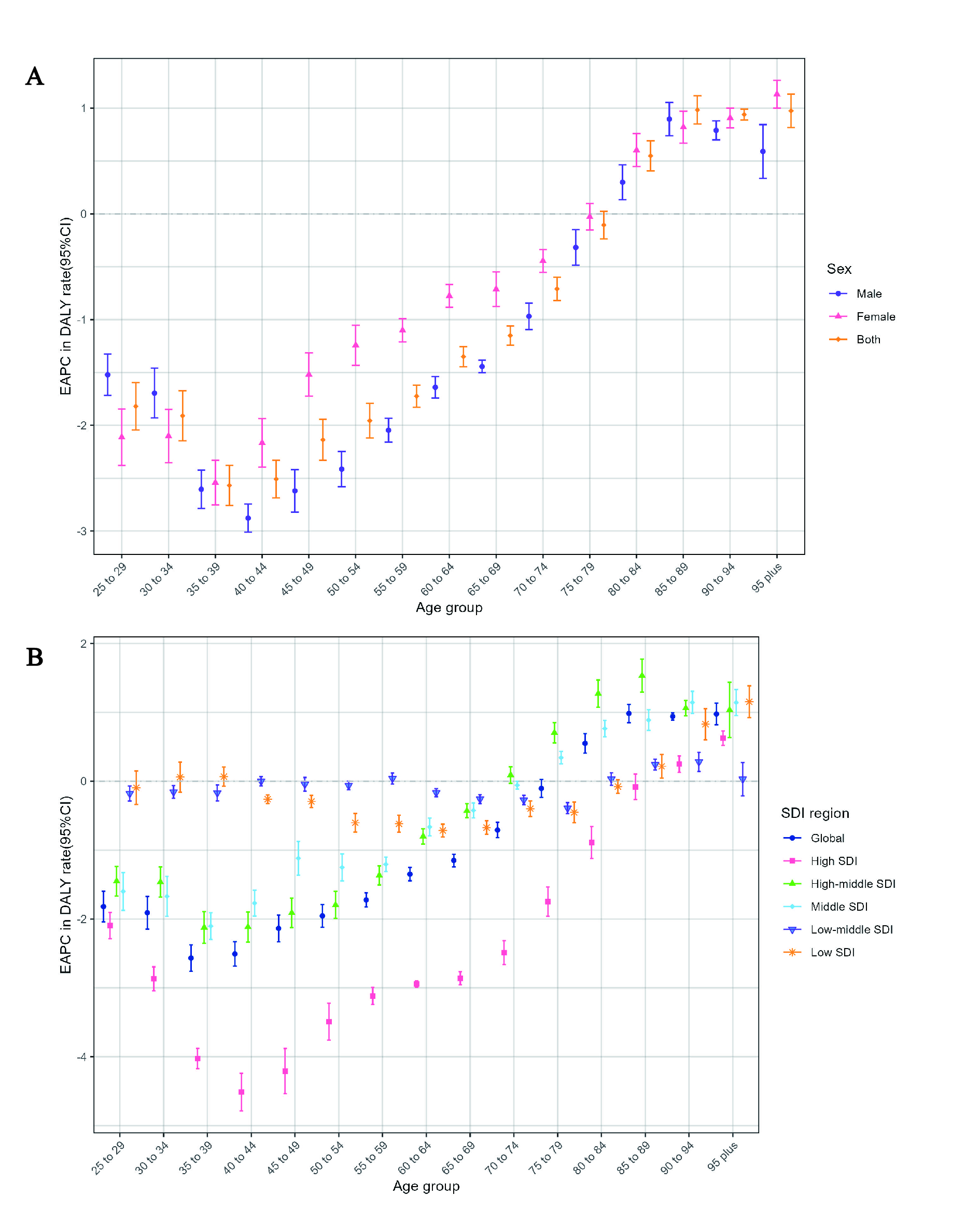


Figure S3. The age distribution of lung cancer EAPC in age-specific DALY rate attributable to second-hand smoke by sex (A) and SDI region (B) from 1990 to 2021. EAPC, estimated annual percentage change; DALYs, disability-adjusted life-year; SDI, sociodemographic index.


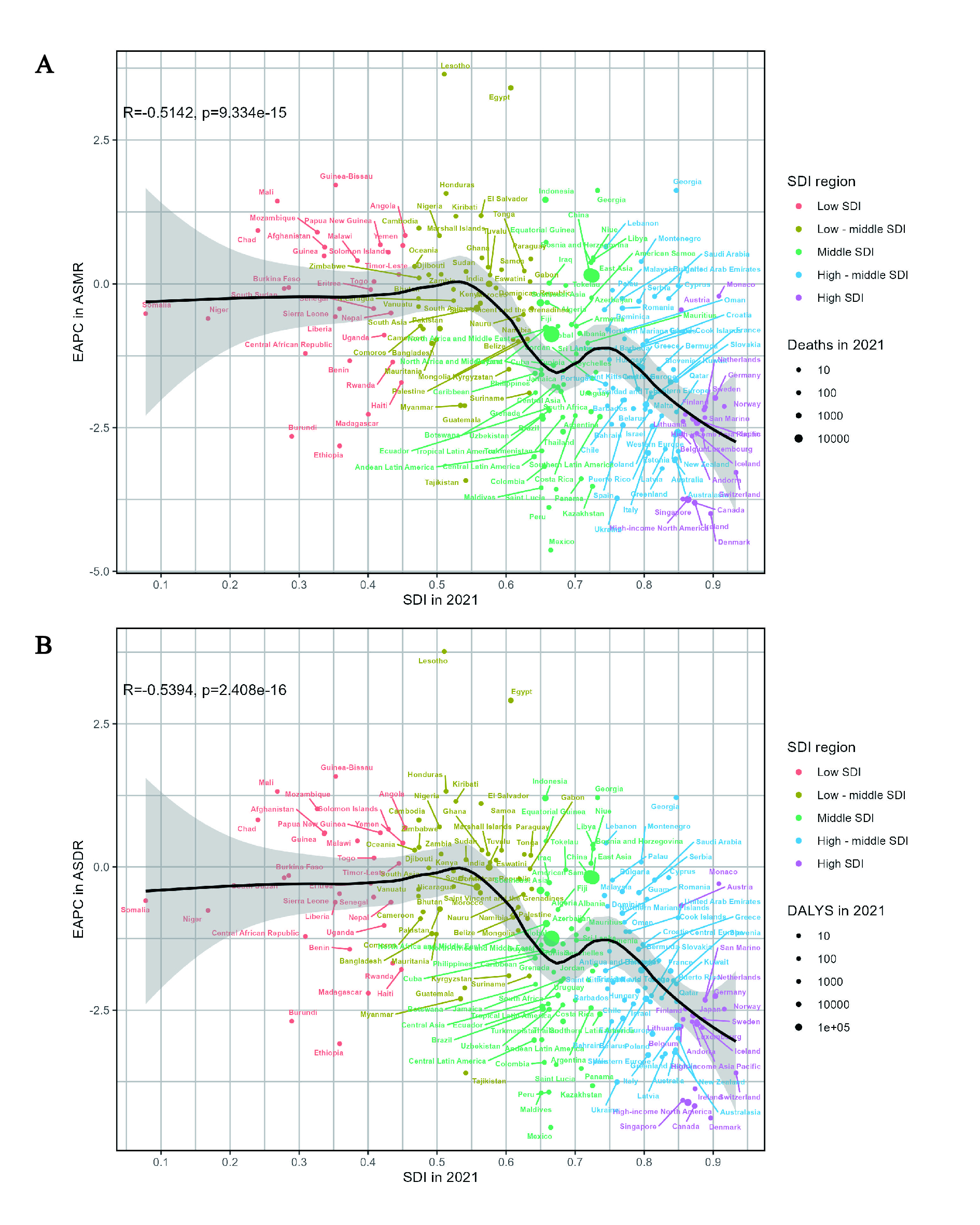


Figure S4. The associations between EAPC of ASMR (A) and ASDR (B) for lung cancer attributable to second-hand smoke and SDI by 204 countries in 2021. ASMR, age-standardized mortality rate; ASDR, age-standardized DALYs rate; DALYs, disability-adjusted life-year; SDI, sociodemographic index.


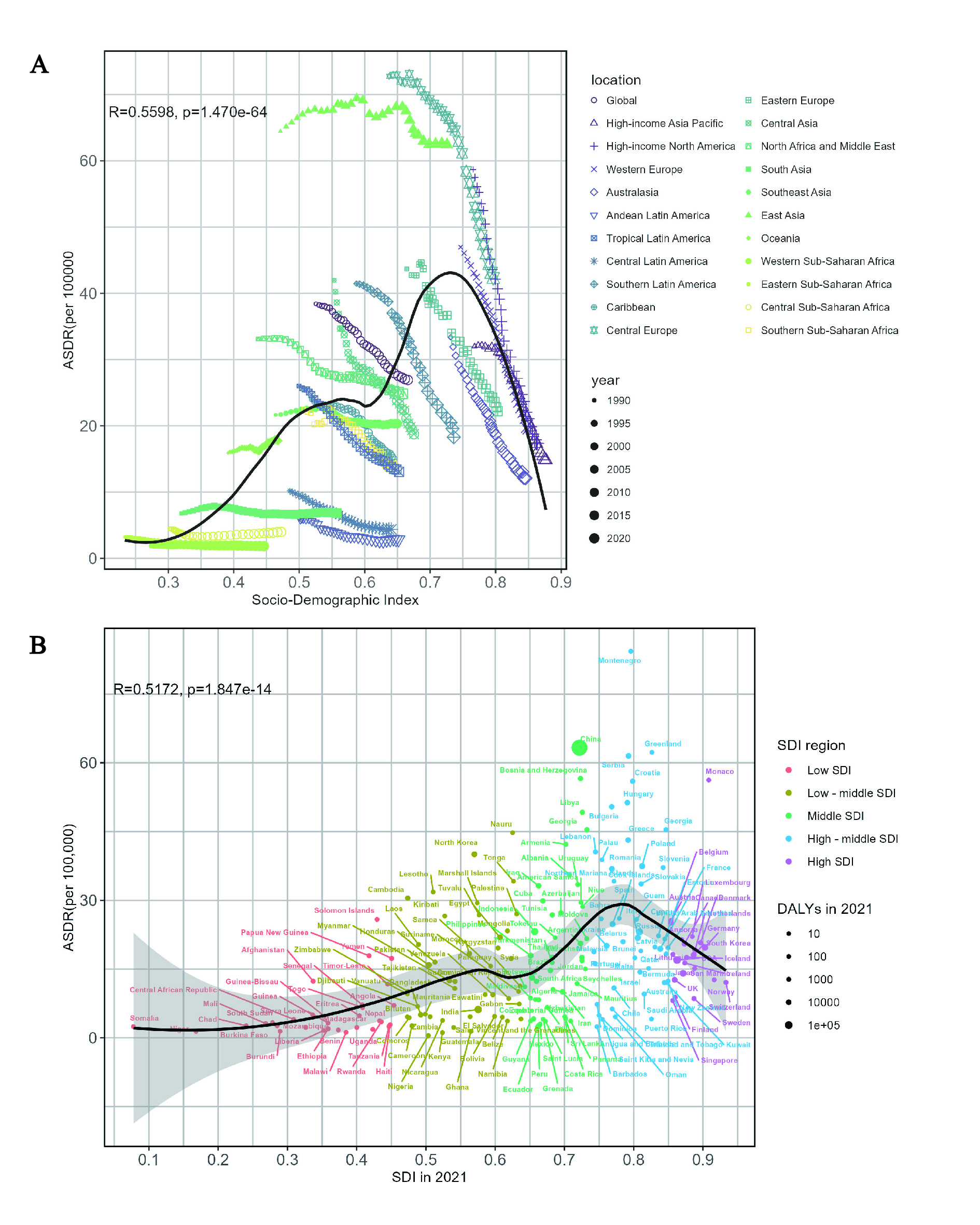


Figure S5. The associations between lung cancer ASDR attributable to second-hand smoke and SDI by 22 regions (A) and 204 countries (B) in 2021. ASDR, age-standardized DALY rate; SDI, sociodemographic index.
